# Diversity and Distribution of Freshwater Testate Amoebae (Protozoa) Along Latitudinal and Trophic Gradients in China

**DOI:** 10.1007/s00248-014-0442-1

**Published:** 2014-06-10

**Authors:** Lihua Ju, Jun Yang, Lemian Liu, David M. Wilkinson

**Affiliations:** 1 Aquatic EcoHealth Group, Key Laboratory of Urban Environment and Health, Institute of Urban Environment, Chinese Academy of Sciences, Xiamen, 361021 People’s Republic of China; 2 University of Chinese Academy of Sciences, Beijing, 100049 People’s Republic of China; 3 School of Natural Science and Psychology, Liverpool John Moores University, Byrom Street, Liverpool, L3 3AF UK

## Abstract

Freshwater microbial diversity is subject to multiple stressors in the Anthropocene epoch. However, the effects of climate changes and human activities on freshwater protozoa remain poorly understood. In this study, the diversity and distribution of testate amoebae from the surface sediments were investigated in 51 Chinese lakes and reservoirs along two gradients, latitude and trophic status. A total of 169 taxa belonging to 24 genera were identified, and the most diverse and dominant genera were *Difflugia* (78 taxa), *Centropyxis* (26 taxa) and *Arcella* (12 taxa). Our analysis revealed that biomass of testate amoebae decreased significantly along the latitudinal gradient, while Shannon-Wiener indices and species richness presented an opposite trend (*P* < 0.05). The relationship of diversity and latitude is, we suspect, an artifact of the altitudinal distribution of our sites. Furthermore, biomass-based Shannon-Wiener index and species richness of testate amoebae were significantly unimodally related to trophic status (*P* < 0.05). This is the first large-scale study showing the effects of latitude and trophic status on diversity and distribution of testate amoebae in China. Therefore, our results provide valuable baseline data on testate amoebae and contribute to lake management and our understanding of the large-scale global patterns in microorganism diversity.

**Electronic supplementary material:**

The online version of this article (doi:10.1007/s00248-014-0442-1) contains supplementary material, which is available to authorized users.

## Introduction

Freshwater is central to the environmental sciences, and as Moss rightly states, it is impossible to overestimate its importance [47]. Lakes and reservoirs are especially important resources for the environmental quality, economic development and social well-being in both developing and developed countries [3, 47]. However, natural or human-induced declines in freshwater biodiversity have influenced ecosystem processes and decreased ecosystem services, thereby diminishing human well-being [20]. A better understanding how climate changes and human activities affect freshwater ecosystems is needed for the effective management and restoration of lake ecosystems and the estimation of their responses to global change [17, 25]. In such studies, it is important to choose representative biotic proxies and environmental parameters to help quantify the state of the system. Fortunately, lake sediments contain a natural archive of remains of various organisms and therefore offer an excellent potential for investigating the impact of climate or human on aquatic ecosystems [68].

For lake ecosystems, diatoms and chironomids have been successfully used as biotic proxies for the reconstruction and assessment of past environmental conditions [9, 29, 38, 43]. Less well known are the more limited studies using testate amoebae as bioindicators to test long-term environment changes [18, 36, 45, 50, 61]. Testate amoebae are a polyphyletic group of unicellular protozoa which are characterized by a decay-resistant and morphologically distinctive test [6]. Although polyphyletic, they form a functional grouping of organisms with broadly similar ecologies [67], being an important component of aquatic ecosystems and playing a significant biogeochemical and ecological role in both terrestrial and freshwater ecosystems [45, 87]. Due to their rapid population growth rates, high abundance and diversity, well preserved tests and most importantly environmental sensitivity, testate amoebae have recently been increasingly used as biotic proxies of environmental change [45, 57, 76]. To successfully use testate amoebae as environmental indicators of anthropogenic global change, we first need to understand the large-scale natural patterns in their distribution—here, we focus on latitudinal gradients and trophic status of testate amoebae in lakes and reservoirs across China.

The latitudinal gradient of species richness remains one of the most prominent patterns in macroecology and biogeography [56, 58, 81]. Clearly, latitude itself is part of an artificial grid system; however, it does nicely summarize the empirical observation that species diversity in many groups tends to decline as one moves away from the tropics. Thus, latitude is a useful surrogate for a whole range of features of the environment which is often used in biogeographical analyses [64]. It has been long established that the diversity of plant species and many animal taxa decreases with increasing latitude. However, there are a few exceptions to this general pattern, and the patterns may be dependent on characteristics of spatial scale and taxonomic hierarchy [81]. Thus, there is an ongoing debate on the underlying mechanisms driving the commonly observed decline of diversity along latitudinal gradient [34, 58, 64]. Although well established for many groups of macroorganisms, it has been unclear if this classic pattern of high species richness at low latitudes could apply to micrcoorganisms [64]. For example, there is a lack of such a relationship for the well-studied diatoms [31]. More recently, however, such relationships have been described for some groups of microorganisms such as freshwater phytoplankton [70] and planktonic marine bacteria [24]. The ecology of testate amoebae has been extensively studied in wetlands and terrestrial soils [45, 74, 80], and there is published evidence that testate amoebae species decreased towards high southern latitudes in continental Antarctica [66]. However, Sherratt and Wilkinson questioned if this relationship was robust as more recent data from some of the high-latitude sites used by Smith [66] has greatly increased the number of recorded taxa [64, 65]. However, it seems clear that at least some testate amoebae groups are largely restricted to tropical and subtropical habitats. For example, *Hoogenraadia* [8] is a largely tropical genus which suggests the possibility of a peak in tropical diversity for testate amoebae. In addition, an increase of biomass with decreasing latitude was found in a European forest transect [62]. However, to our knowledge, there was still no report on testate amoebae biomass in lake sediment along a latitudinal gradient. In this study, we hypothesized that the abundance, biomass, species richness and diversity of testate amoebae were negatively related to the latitude. We tested this hypothesis by conducting a comparative study of testate amoebae communities along a latitudinal gradient ranging from about 23° to 50° N from surface sediments in 51 lakes and reservoirs of China.

The relationships between testate amoebae communities and lake trophic status have long been studied [21, 60, 72, 86]. However, the testate amoebae in the Eastern Hemisphere, in contrast to the western, have been less extensively investigated. In particular, previous records of testate amoebae in lakes from China are few and scattered, and most of these studies sampled lake waters—so only sampling planktonic testate amoebae at a particular time of year [7, 55, 63, 85–88]. Recently, Yang et al. concluded that trophic status was a main factor that governed the composition and distribution of testate amoebae in Yunnan lakes [86]. It has been widely reported that certain testate amoebae species exhibit preferences for different eutrophic levels [5, 55, 73]. Nonetheless, variable responses of testate amoebae to trophic status are common, because they also respond to other environmental factors, such as other aspects of lake water chemistry, habitat heterogeneity and invertebrate predation [21, 30, 36, 75]. Further, most studies dealing with testate amoebae communities have focused on freshwater ecosystems located in restricted and narrow latitude or climate ranges. Since early surveys pointed to a preponderance of unimodal relations between trophic status and plankton [33], we hypothesized that the abundance, biomass, species richness and diversity of testate amoebae may present bell-shaped curves with trophic state level being minimum at the oligotrophic and hypereutrophic levels. We test this hypothesis by identifying and comparing the patterns of responses of testate amoebae communities to trophic indices across a large geographic scale covering a variety of lakes and reservoirs in temperate and subtropical regions.

In the opening of a classic mid-twentieth century paper, Hutchinson wrote ‘The great intellectual fascination of limnology lies in the comparative study of a great number of systems, each having some resemblance to the others and also many differences’ [32]. Following Hutchinson’s lead, the aims of this study were (1) to characterize testate amoeba communities from surface sediments in 51 lakes and reservoirs of China and (2) to test whether the species diversity and distribution of testate amoebae were significantly related to latitude and trophic status, respectively, or largely driven by more local lake-specific factors. By sampling surface sediments, we can collect data on both benthic testate amoebae and planktonic forms—which have sunk to the bottom of the lake—and such a sample gives a time-averaged description of the testate community over a timescale of a small number of years.

## Materials and Methods

### Study Area and Sampling

**Fig. 1:**
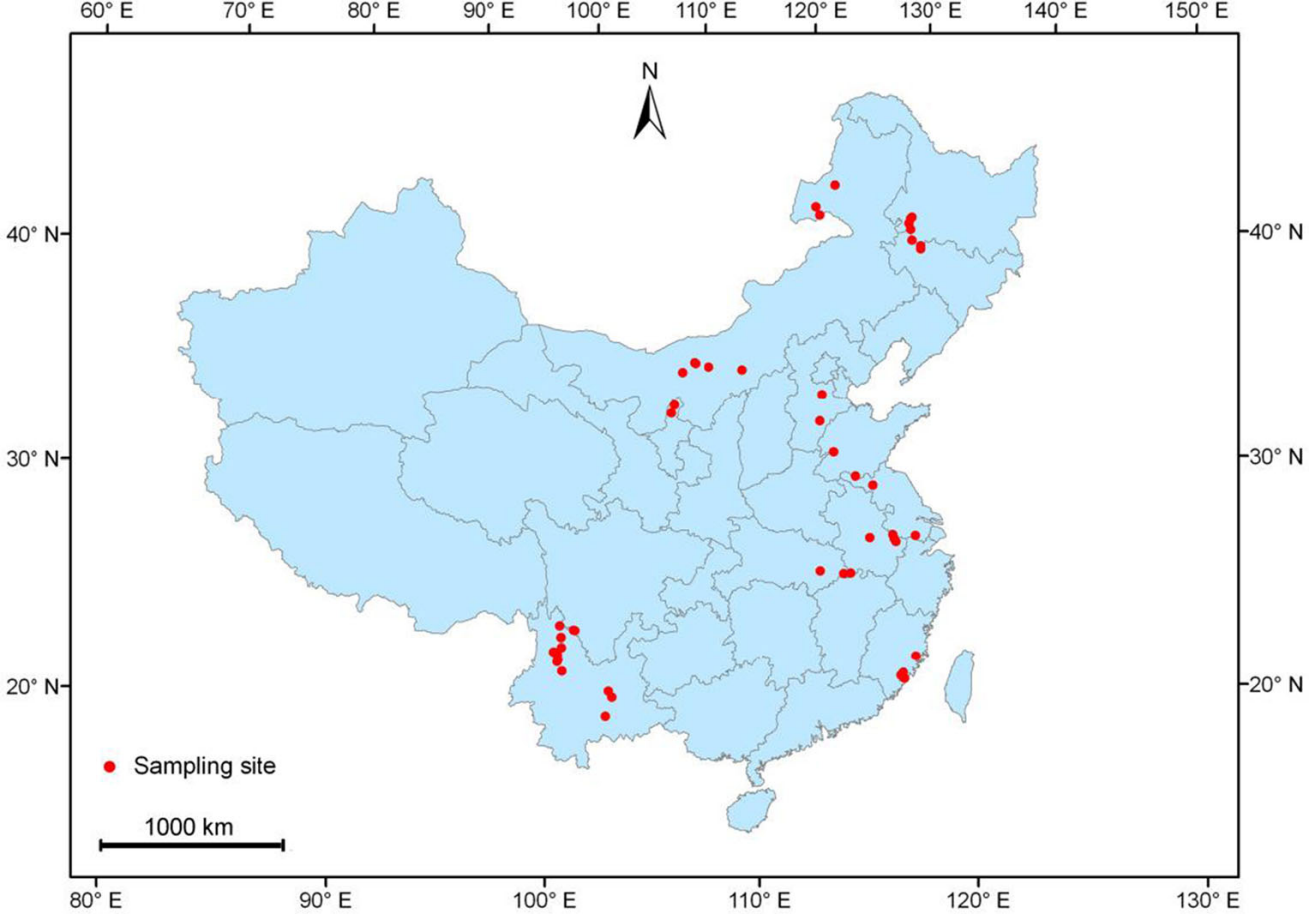
Location of the 51 study lakes and reservoirs in China

During the summer of 2012, a total of 51 lakes and reservoirs across China were selected for testate amoebae analysis along a latitudinal gradient, ranging from approximately 23° to 50° N (Table 1, Fig. 1). The altitude of these lakes and reservoirs varied from 0 to 4,231 m. This study area includes several major climate types, namely subtropical monsoon climate, temperate monsoon climate and plateau climate. Mean annual temperature in east China ranges from −1.00 °С in the northeast to 21.00 °С in the southeast, and total annual precipitation increases from approximately 300 mm to more than 1,600 mm [77]. There is a large seasonal temperature variation (mean temperature between July and January) range (9.50–53.00 °C) in different lakes and reservoirs along latitudinal gradient.

**Table 1:** List of the 51 study lakes and reservoirs in China

Lake code	Lake name	Region	Trophic state	Latitude (° N)	Longitude (° E)
1 YLL	Yilong L.	YN	HEU	23.6717	102.5892
2 HBR	Hubian R.	FJ	LEU	24.4972	118.1536
3 FXL	Fuxian L.	YN	OM	24.5676	102.8882
4 BTR	Bantou R.	FJ	LEU	24.6747	118.0214
5 SDR	Shidou R.	FJ	LEU	24.6925	118.0092
6 TXR	Tingxi R.	FJ	MES	24.8031	118.1392
7 DCL	Dianchi L.	YN	HEU	24.8566	102.7039
8 DZR	Dongzhen R.	FJ	LEU	25.4839	118.9434
9 EHL	Erhai L.	YN	LEU	25.7334	100.2157
10 CBL	Cibi L.	YN	MES	26.1705	99.9397
11 HXH	Haixihai L.	YN	MES	26.2871	99.9671
12 JHL	Jianhu L.	YN	MES	26.4865	99.9300
13 SML	Shengmu L.	YN	MES	26.6289	99.7091
14 ZML	Zimei L.	YN	OM	26.6319	99.7115
15 TCL	Tiancai L.	YN	OM	26.6343	99.7168
16 LSH	Lashi L.	YN	MES	26.8803	100.1283
17 HBHU	Habahuang L.	YN	OM	27.3465	100.0718
18 HBHE	Habahei L.	YN	OM	27.3554	100.0701
19 LGUL	Lugu L.	YN	OL	27.7167	100.8000
20 XHZ	Xiaohaizi L.	YN	MES	27.7403	100.7200
21 SDL	Shudou L.	YN	MES	27.9105	99.9506
22 LGAL	Longgan L.	CJ	MEU	29.9361	116.1661
23 TBL	Taibai L.	CJ	HEU	29.9555	115.7979
24 LZL	Liangzi L.	CJ	HEU	30.2405	114.5122
25 NYL	Nianyi L.	CJ	MEU	31.1190	118.9753
26 TAL	Taihu L.	CJ	HEU	31.2199	120.1409
27 GCL	Gucheng L.	CJ	MEU	31.2761	118.9220
28 SJL	Shijiu L.	CJ	LEU	31.4740	118.8879
29 CHL	Chaohu L.	CJ	MEU	31.5193	117.5583
30 LML	Luoma L.	EC	MES	34.0534	118.2205
31 WSL	Weishan L.	EC	MEU	34.6388	117.2817
32 DPL	Dongping L.	EC	MEU	35.9686	116.1921
33 HSL	Hengshui L.	EC	MEU	37.6199	115.6251
34 YHL	Yuehai L.	IM	MEU	38.5618	106.2040
35 BYD	Baiyangdian L.	EC	HEU	38.9432	115.9826
36 XHL	Xinghai L.	IM	MEU	38.9857	106.4048
37 HSH	Hasuhai L.	IM	MEU	40.6109	110.9715
38 DHZ	Donghaizi L.	IM	OM	40.6308	107.0031
39 WLSH	Wuliangsuhai L.	IM	LEU	40.8685	108.7931
40 QSHZ	Quansanhaizi L.	IM	LEU	41.0679	107.8689
41 SLHZ	Shenglihaizi L.	IM	MEU	41.1225	107.8273
42 XMP	Xinmiaopao L.	NE	HEU	45.2120	124.4460
43 KLP	Kulipao L.	NE	MEU	45.3711	124.4967
44 YLP	Yueliangpao L.	NE	MEU	45.7380	124.0030
45 LMSP	Lamasipao L.	NE	HEU	46.2915	124.0954
46 AMTP	Amutapao L.	NE	HEU	46.6081	124.0612
47 QJP	Qijiapao L.	NE	MEU	46.8240	124.2779
48 TIL	Tianhu L.	NE	HEU	46.8737	124.4015
49 BEL	Bei’er L.	IM	LEU	47.9336	117.7000
50 WLP	Wulanpao L.	IM	LEU	48.3609	117.5229
51 HHNE	Huhenuo’er L.	IM	MEU	49.2960	119.2344

*YN* Yunnan, Southwest China, *CJ* the middle and lower reaches of the Yangtze River Valley, China, *EC* East Central China, *FJ* Fujian, Southeast China, *IM* Inner Mongolia region, North China, *NE* Northeast China, *OL* oligotrophic, *OM* oligo-mesotrophic, *MES* mesotrophic, *LEU* light eutrophic, *MEU* middle eutrophic, *HEU* hypereutrophic

No specific permits were required for sampling these lakes or reservoirs for the described field studies. The latitude and longitude coordinates and altitude for the sampling sites were determined using a portable global positioning system (GPS) (Jisibao G330, Beijing, China). At each lake or reservoir, triplicate sediment samples within 10–50 m were collected from the deepest area using a Kajak sediment corer from a small boat. The upper 1 cm of the sediments from the triplicate samples was mixed in the field and processed for testate amoebae remains, because the top 0–1-cm sediment represents an integrated sample of the entire lake’s production over the past 1–4 years [68]. Sediments were subsequently stored in polyethylene bags at 4 °С in the dark. All samples were returned to the laboratory as soon as possible (within 1 day) after fieldwork.

### Testate Amoebae Analysis

In the laboratory, all sediment samples were preserved in a dark refrigerator with temperature at 4 °С to prevent the infections from fungi and bacteria until analysis [6]. For each lake sample, 1 cm^3^ of homogenized surface sediments was subsampled and placed in a clean glass beaker containing distilled water, and subsequently stirred gently for 5 min to separate mineral particles from the shells. The sample was then sieved on meshes of 300 and 25 μm to remove the large particles and the fine organic and mineral detritus. All fractions between 300 and 25 μm were washed into a brown bottle and diluted to 50 ml with distilled water. Both living and dead testate amoebae were examined in Hydro-Bios plankton chambers under an inverted microscope at ×200–400 magnification, and all measurements of shell length or diameter were taken using an ocular micrometre. Considering the aim of this study, relatively minor, but still potentially ecologically relevant community changes need to be identified; thus, at least 150 living and dead individuals were counted for each sample [52]. Identification of the specimens was based on the classic literature [13–16, 37, 41, 42, 49, 53, 63]. All specimens were identified down to the lowest possible taxonomic level except when insufficient taxonomic characters could be observed.

### Limnological Analysis

Water transparency was estimated in situ with a 30-cm diameter Secchi disc. Water depth of sampling site was measured with a Speedtech SM-5 Depthmate portable sounder (Speedtech Instruments, USA). The depth-integrated water samples were collected from the centre of the lakes or reservoirs for analysis of chlorophyll a, total nitrogen and total phosphorus concentrations and measured according to standard methods [26]. Water temperature of the epilimnion layer was measured using a multi-parameter water quality analyzer (YSI, USA) every metre from the surface to bottom waters, and the mean value was recorded and used in this study. The comprehensive trophic state index (TSI) was calculated using the above-mentioned limnological parameters, including chlorophyll a (μg l^−1^), Secchi disc transparency (m) and total phosphorus (TP, μg l^−1^) [11]. The comprehensive TSI ranks different trophic state conditions of lakes on a numerical scale of 0 to 100: 0 < TSI ≤ 30 oligotrophic, 30 < TSI ≤ 40 oligo-mesotrophic, 40 < TSI ≤ 50 mesotrophic, 50 < TSI ≤ 60 light eutrophic, 60 < TSI ≤ 70 middle eutrophic and 70 < TSI ≤ 100 hypereutrophic [11, 12, 84].

### Numerical Analysis

The testate amoebae data were expressed as both abundance and biomass. The abundance was calculated based on counted testate amoebae numbers as the actual number of 1-ml sediment. The carbon biomass for each species was estimated based on the shell geometric volume using our size measurements and calculated using the following carbon/volume conversion factor: 1 μm^3^ = 1.1 × 10^−7^ μg C [78]. The total biomass of testate amoebae in each lake or reservoir was a sum of all individuals. To explore the testate amoebae community variation along latitudinal and trophic gradients, five biological community parameters (i.e. abundance, biomass, abundance-based Shannon-Wiener index, biomass-based Shannon-Wiener index and species richness) in each sample were calculated, respectively. The dominance value (*Y*) of each species in each lake was obtained (ratio of the abundance of certain morphospecies with total abundance in each lake) [82], and taxa with a dominance value of more than 0.02 in each sample were defined as dominant species of this lake. The Shannon-Wiener index was calculated on the basis of abundance and biomass for each sample to examine the species diversity because the calculations based on biomass may be the best descriptors for the studied lakes and reservoirs [23, 50, 69]. The biotic data and environmental variables were log(x + 1) transformed to improve normality and homoscedasticity before multivariate statistical analysis. Detrended correspondence analysis (DCA) was applied to determine the gradient length. Then, the canonical correspondence analysis (CCA) was selected to determine the environmental variables that had a greater influence on testate amoebae assemblages as the gradient lengths were >3 standard deviations (4.0 SD for axis 1). Forward selection and Monte Carlo permutation tests (999 permutations under a full model) were used to test the contribution and significance of each variable. Partial canonical correspondence analysis (pCCA) was explored to establish the marginal effect of each variable (pCCA run individually with each environmental variable without the forward selection procedure). Further, both redundancy analysis (RDA) and partial redundancy analysis (pRDA) were also performed on Hellinger-transformed abundance and biomass data to better highlight discontinuities in communities. Moreover, RDA and pRDA were run on both *Difflugia* abundance matrix and the matrix without *Difflugia* species since *Difflugia* species were dominant in many lakes and potentially could mask some important information explained by less abundant species. All the above analysis were performed using CANOCO version 4.5 [71]. The relationships between testate amoebae community parameters and latitude and trophic status gradients were explored with Pearson correlation coefficient (*r*) or Spearman correlation coefficient (*r*_s_)—depending on the data characteristics. The data analysis was performed using PRIMER version 5.0, STATISTICA version 6.0 and SPSS version 19.0.

## Results

### Testate Amoebae Community

**Fig. 2:**
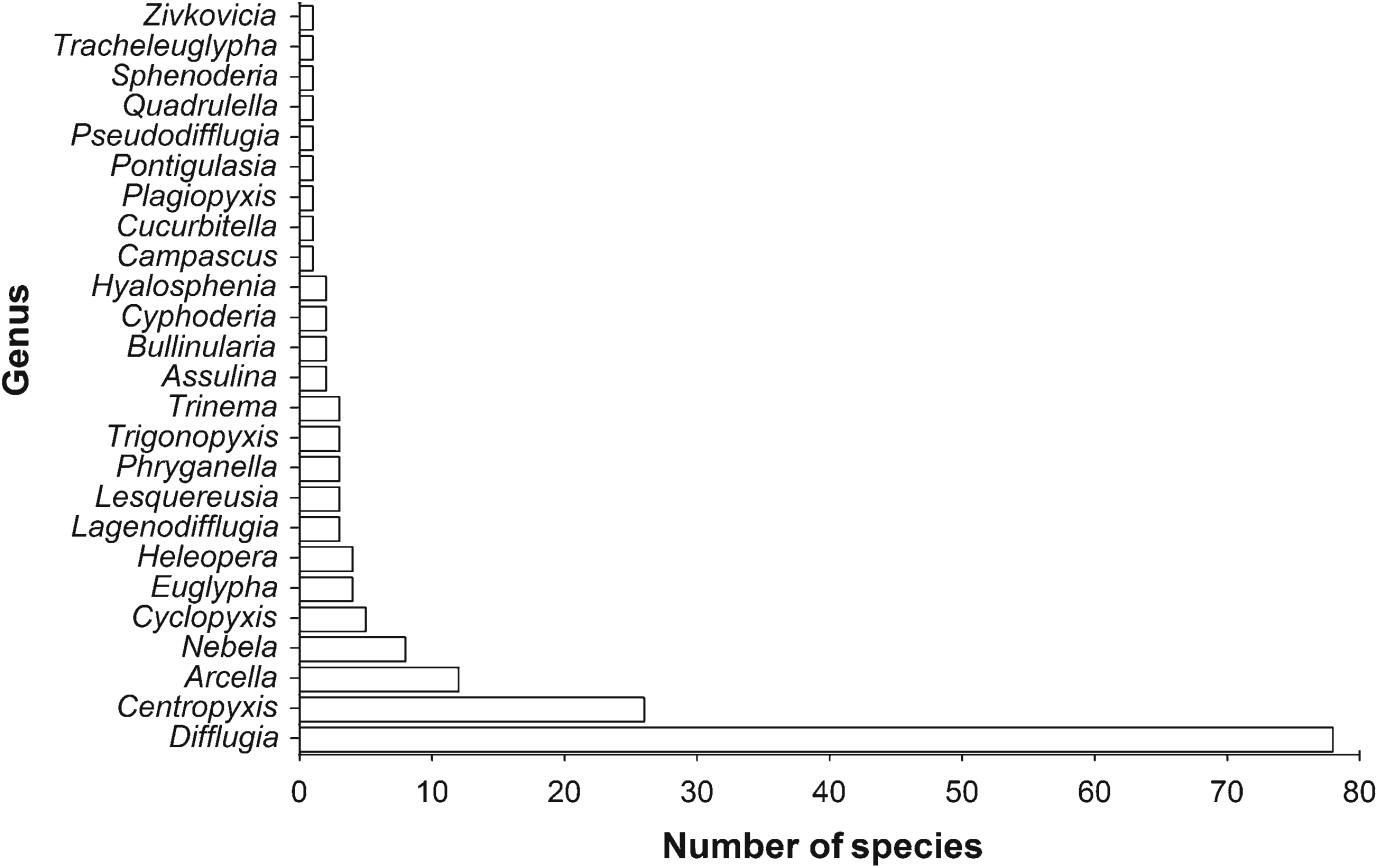
Number of testate amoebae taxa per genus in 51 lakes and reservoirs in China

In total, 169 testate amoebae taxa belonging to 24 genera were identified in the surface sediments of the 51 lakes and reservoirs. The majority of these species (154 taxa) belonged to order Arcellinida, Kent 1880. Most species and subspecies were in the genera *Difflugia* (78 taxa), followed by *Centropyxi*s (26 taxa) and *Arcella* (12 taxa) (Fig. 2). The species (including subspecies) richness in each lake or reservoir varied greatly; 23.7 % (40 taxa) of all the identified species and subspecies were discovered only in one lake and 10.1 % (17 taxa) in more than half of the lakes. Moreover, the highest-frequency species belonging to genus *Difflugia* (i.e. *Difflugia penardi*) appeared in 49 lakes (96.1 %), and no species were observed in all study lakes and reservoirs (Fig. S1). The dominant species (species with dominance index *Y* > 0.02) differed greatly among the study lakes and reservoirs (Table S1). For instance, *Difflugia limnetica* (*Y* = 0.64) was the commonest of the six dominant species in Lake Dianchi, while Lake Lugu was dominated by ten species with *Difflugia gramen* that was the commonest species (*Y* = 0.26).

The species richness of testate amoebae varied between 7 and 46 taxa with a mean of 31 ± 1 standard error (SE). The testate amoebae abundance in sediment samples ranged from 536 to 10,850 ind. ml^−1^ with a mean of 2,563 ± 339 (SE) ind. ml^−1^, while the carbon biomass varied from 6.03 to 192.53 μg C ml^−1^ with an average of 45.42 ± 6.13 (SE) μg C ml^−1^ (Table 2). Furthermore, the abundance of testate amoebae was positively related to the biomass (*r*_s_ = 0.77, *P* < 0.001, *n* = 51). In addition, both abundance-based and biomass-based Shannon-Wiener indices were significantly positively correlated to species richness at *P* < 0.001, indicating that Shannon-Wiener index and species richness were closely correlated with each other.

**Table 2:** List of the testate amoebae community parameters from the 51 study lakes and reservoirs in China

Lake code	Abundance (ind. ml^−1^)	Biomass (μg C ml^−1^)	Species richness	Abundance-based *H′*	Biomass-based *H′*
YLL	1,408	16.85	28	2.48	2.71
HBR	2,700	55.68	28	2.68	2.33
FXL	1,790	11.25	18	1.69	1.57
BTR	7,840	121.42	30	2.28	2.07
SDR	8,120	146.97	29	2.47	2.28
TXR	1,590	25.74	37	2.97	2.84
DCL	922	18.80	17	1.47	1.39
DZR	835	14.43	25	2.33	2.40
EHL	2,534	78.45	39	3.22	2.87
CBL	2,163	21.19	28	2.33	2.69
HXH	1,185	11.16	14	0.76	1.14
JHL	1,508	44.04	19	2.15	1.69
SML	2,567	157.14	22	2.35	0.85
ZML	2,150	90.31	31	2.78	1.25
TCL	9,500	54.41	39	2.90	3.08
LSH	1,630	33.30	20	2.31	1.81
HBHU	2,717	29.68	24	2.27	2.50
HBHE	1,450	48.49	29	2.71	1.89
LGUL	599	6.69	32	2.78	2.91
XHZ	2,917	31.90	24	2.08	1.96
SDL	655	96.25	42	3.18	1.19
LGAL	10,850	146.06	39	3.12	3.07
TBL	1,425	25.14	32	2.65	2.71
LZL	1,850	37.37	28	2.60	2.44
NYL	3,750	53.41	38	2.90	2.95
TAL	1,920	20.00	30	2.10	2.51
GCL	6,225	100.53	40	2.85	2.84
SJL	8,375	192.53	40	2.82	2.30
CHL	4,125	85.00	24	1.82	1.72
LML	989	17.53	37	2.78	2.97
WSL	536	6.03	35	3.09	3.12
DPL	2,634	61.11	37	3.14	2.40
HSL	2,025	35.54	40	3.31	2.69
YHL	795	10.67	34	3.16	2.85
BYD	3,500	67.46	34	2.82	2.67
XHL	1,620	11.78	31	2.96	3.09
HSH	1,900	24.50	27	2.44	2.72
DHZ	4,300	18.73	7	0.59	0.90
WLSH	667	10.10	34	2.51	2.78
QSHZ	2,584	31.71	33	2.79	2.76
SLHZ	970	7.05	24	2.31	2.64
XMP	2,175	40.96	46	3.34	3.11
KLP	557	7.64	41	3.26	3.07
YLP	2,400	51.55	38	2.92	2.77
LMSP	906	7.69	24	2.48	2.71
AMTP	607	17.42	33	3.09	1.83
QJP	2,120	33.07	46	3.22	2.89
TIL	1,186	23.66	21	2.34	1.30
BEL	1,350	22.34	38	3.01	3.01
WLP	591	11.55	38	3.21	2.98
HHNE	1,002	13.10	46	3.46	3.38

*H′* Shannon-Wiener index

**Fig. 3:**
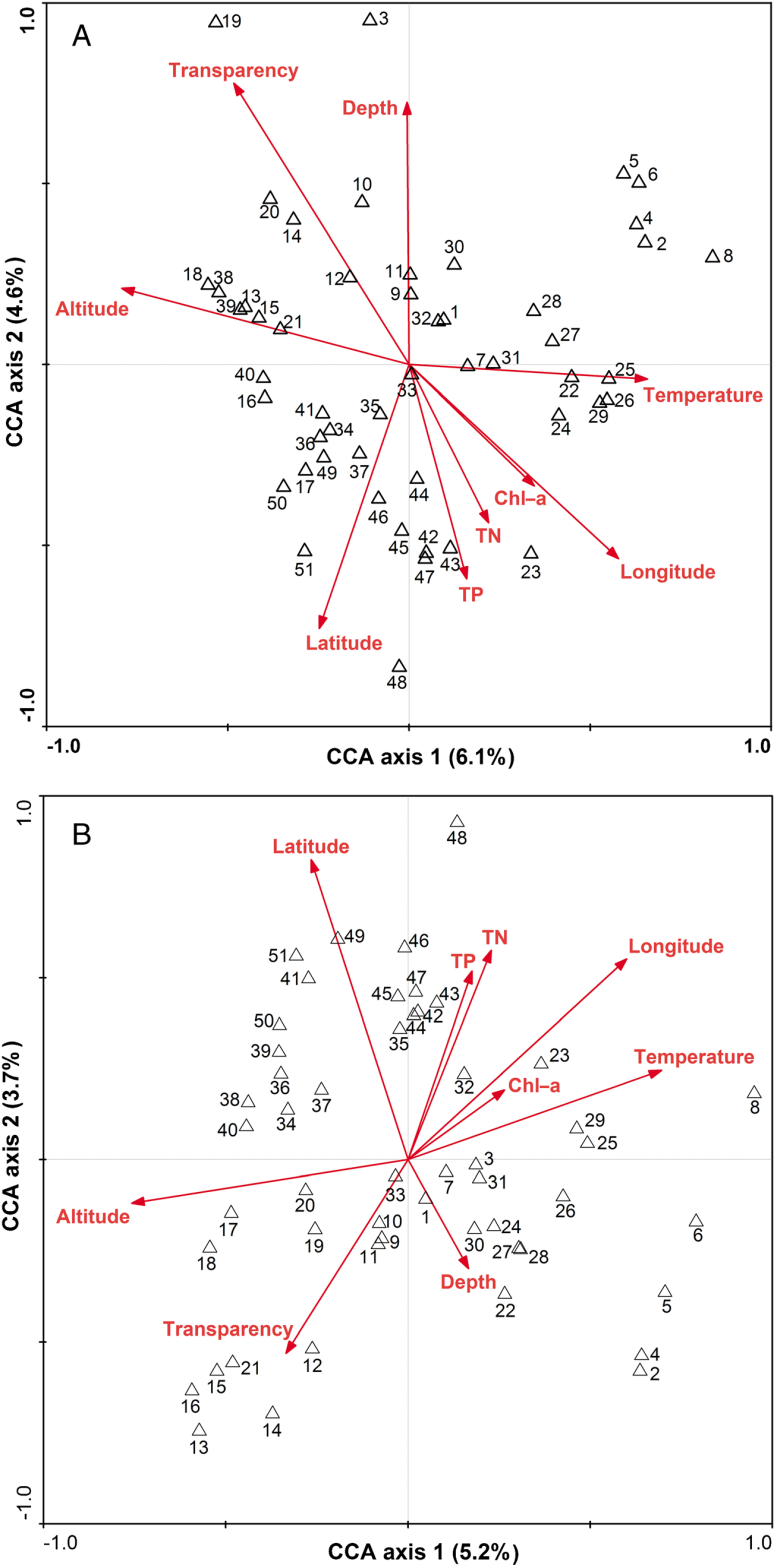
Canonical correspondence analysis (CCA) sample-environment biplot for the 51 lakes and reservoirs that yield statistically significant testate amoebae populations. **a** Abundance, **b** biomass. The sample sites are given in Table 1

All variables explained 6.1 % of the variance for abundance-based communities in CCA axis 1, while 4.6 % in axis 2 (Fig. 3a). The full CCA with forward selection of the environmental variables showed that the geographical variables (altitude, latitude and longitude) were significant (*P* < 0.001) in affecting the testate amoebae assemblages. However, the marginal effect of each variable based on pCCA showed that latitude was significant at *P* < 0.01 in structuring the testate amoebae assemblages, whereas longitude (*P* = 0.023) and altitude (*P* = 0.017) were significant at *P* < 0.05 (Table S2). The CCA results based on biomass-based communities were similar to CCA results based on abundance-based communities (Fig. 3b). The full CCA with forward selection of the environmental variables also showed that latitude and longitude were significant (*P* < 0.001) in affecting the testate amoebae assemblages. The results of pCCA showed that latitude and water depth were significant at *P* < 0.05 in structuring the testate amoebae communities (Table S2).

Compared to CCA results, more variance was explained in RDA, but both RDA based on abundance and biomass showed that latitude was significant (*P* < 0.05) in affecting the testate amoebae assemblages similar as CCA results (Fig. S2a, b). Moreover, both RDA and pRDA results of abundance-based data with only *Difflugia* species and without *Difflugia* species also emphasized the important effect of latitude (*P* < 0.05) (Fig. S2c, d, Table S3). Additionally, the limnological variables chlorophyll a and total nitrogen (TN) explained 5.1 and 4.4 % of the variance for *Difflugia* species while temperature and transparency explained 4.5 and 3.7 % of the variance for abundance-based communities without *Difflugia* species, respectively.

### Testate Amoebae Along the Latitudinal Gradient

**Fig. 4:**
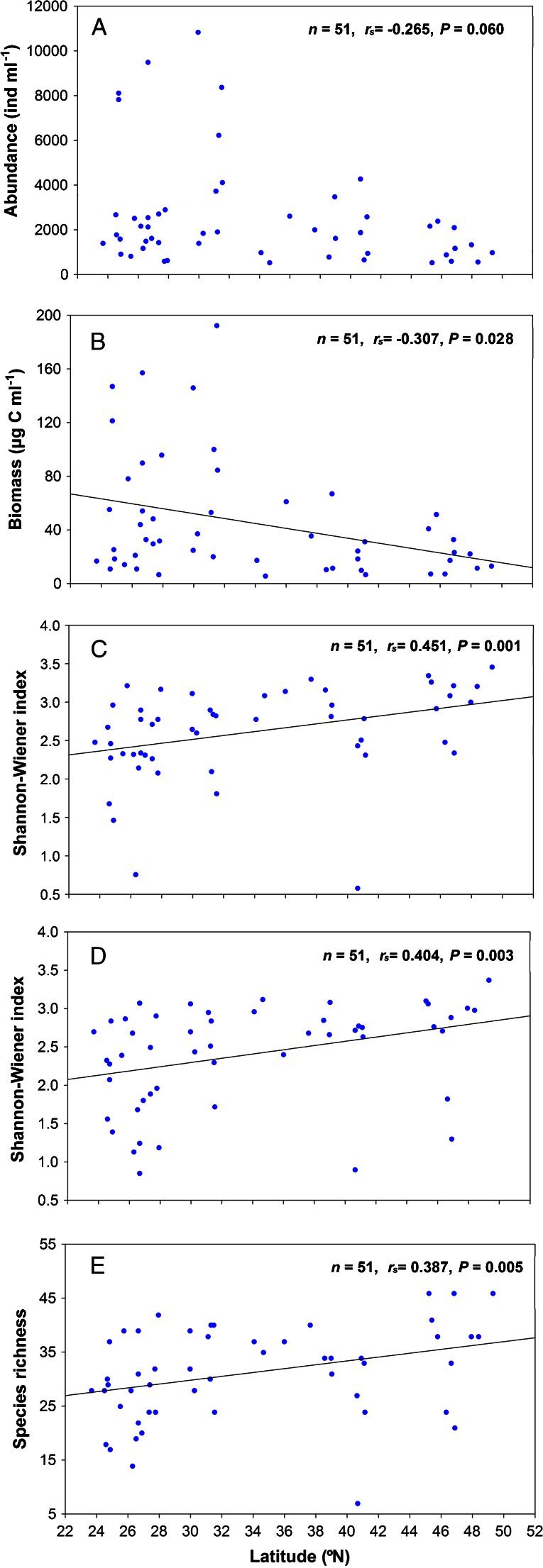
Variation of testate amoebae community parameters along a latitudinal gradient. **a** Abundance, **b** biomass, **c** Shannon-Wiener index based on abundance data, **d** Shannon-Wiener index based on biomass data and **e** species richness

Significant effects of latitude on four of the testate amoebae community parameters (biomass, abundance-based Shannon-Wiener index, biomass-based Shannon-Wiener index and species richness) were detected in this study (Fig. 4b–e), while abundance expressed a marginally significant relationship with latitude (*P* = 0.060) (Fig. 4a). The biomass of testate amoebae showed a significant downward trend with the increasing of latitude (*P* < 0.05). The highest values of abundance (10,850 ind. ml^−1^) and biomass (192.53 μg C ml^−1^) were, respectively, detected in Lakes Longgan and Shijiu, which were approximately located at 29°–32° N. However, Shannon-Wiener index and species richness of testate amoebae exhibited a reversed trend with peaks at the high-latitudinal lakes. When lakes of a similar altitude (all those below 150 m) were compared, the same decreasing trend in abundance and biomass (both *P* < 0.05, *n* = 25) and increasing trend in abundance-based Shannon-Wiener index in relation to increasing latitude (*P* < 0.05, *n* = 25) were found (Fig. S3). However, neither biomass-based Shannon-Wiener index (*r*_s_ = 0.129, *P* = 0.539, *n* = 25) nor species richness (*r*_s_ = 0.244, *P* = 0.240, *n* = 25) was significantly correlated with latitude. As an alternative approach, we reassigned all the lakes’ new latitudes based on their altitude values, that is moving high lakes north and low lakes south—using an approximate lapse rate of −0.60 °C per 100 m and a temperature decline of 0.75 °C per degree of latitude [48] to assign the lakes’ new ‘corrected’ latitudes. Given the several mechanisms by which the actual lapse rate can vary from our textbook simplification, this approach should be interpreted with caution [46]; therefore, we do not want to put too much emphasis on this analysis. However, when we tried this approach, the significant correlation between species richness and latitude also vanished (*r*_s_ = −0.027, *P* = 0.850, *n* = 51), as did relationships with the abundance-based Shannon-Wiener index and the biomass-based Shannon-Wiener index (*P* = 0.581 and *P* = 0.847, respectively) (Fig. S4). Lake Donghaizi was a special lake which located in the higher latitude (40.63° N), and only seven species were identified from it in this study—since it was dominated by *Arcella hemisphaerica*, abundance-based Shannon-Wiener index was the minimum, and biomass-based Shannon-Wiener index was also low (Table 2). The highest species richness (46 taxa) occurred in three lakes located in the higher latitude about 45°–50° N (Lakes Xinmiaopao and Qijiapao in Northeast China and Lake Huhenuo’er in Inner Mongolia, North China).

### Testate Amoebae Along the Trophic Gradient

**Fig. 5:**
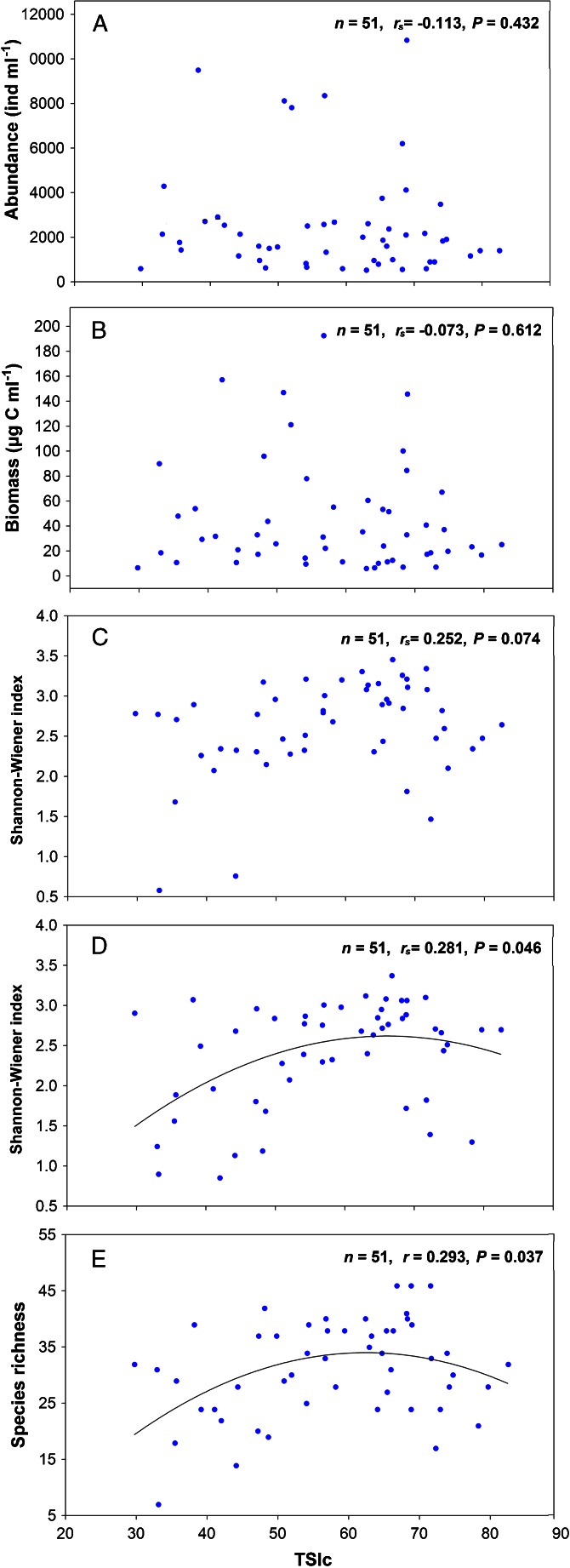
Variation of testate amoebae community parameters along a trophic status gradient. **a** Abundance, **b** biomass, **c** Shannon-Wiener index based on abundance data, **d** Shannon-Wiener index based on biomass data and **e** species richness

In this study, the comprehensive trophic state index (TSIc) ranged from 29.7 in Lake Lugu to 82.5 in Lake Taibai, that is the studied lakes were subjected to different trophic status from oligotrophy to hypereutrophy. Neither abundance nor biomass was significantly correlated with the trophic status, but biomass-based Shannon-Wiener index and species richness both displayed a bell-shaped response along the trophic gradient with peaks at middle eutrophic status (Fig. 5). Moreover, abundance-based Shannon-Wiener index manifested a similar response to trophic status with biomass-based Shannon-Wiener index (*P* = 0.074) (Fig. 5c). The three lakes with maximum species richness showed a similar value of trophic status; they were Lakes Huhenuo’er (66.7), Qijiapao (68.8) and Xinmiaopao (71.5). Although Lake Xinmiaopao was defined as hypereutrophic status, it was classified somewhere near the border of middle eutrophy and hypereutrophy according to its TSIc value.

## Discussion

### Community Composition and Diversity

Our study showed that 169 testate amoebae taxa were identified in the surface sediment samples. Comparing to studies carried out in other counties, for example 17 species and 28 strains were identified in 31 lakes from Florida [22], the diversity of testate amoebae in China was relatively high. Although testate amoebae communities were structurally different between lakes and reservoirs, most species and subspecies were in the genera *Difflugia*, *Centropyxis* and *Arcella*, which are consistent with previous studies in plateau lakes in Yunnan and in Tibet, China [63, 85, 86]. Similarly, Qin et al. found that *Difflugia* (21 species, 81 %) and *Centropyxis* (3 species, 11 %) were the most abundant and diverse genera in Lake Zhangdu in central China [55]. Since these patterns were observed in several different studies and areas, it seems likely that *Difflugia* and *Centropyxis* have the highest number of species in lake ecosystems of China. Indeed, the same dominant genera were found in lake deposits in Europe and North America [22, 76] and even in Arctic and Antarctic regions [83]. Recently, Alves et al. also showed that *Difflugia* (41 species), *Arcella* (18 species), *Centropyxis* (11 species) and *Lesquereusia* (10 species) were the most common genera in a lake of Brazil [1].

Furthermore, the typical dominant taxa *D. gramen globulosa*, *D. limnetica* and *D. penardi* in our study (Table S1) have been previously reported to be the typical component of many lake ecosystems [4, 6, 76]. In fact, many of the lake-dwelling testate amoebae species are considered cosmopolitan in their distribution [35]. The typical taxonomic composition of testate amoebae assemblages which were based on extensive sampling and a comprehensive range of trophic status suggests that our findings could be extended to other lake ecosystem beyond the limits of the studied location.

Both CCA and RDA suggested that testate amoebae were responding to a number of environmental variables in the studied lakes and reservoirs, especially geographical factors (varying with both latitude and longitude and altitude). However, the relatively small size of these effects also confirmed the importance of other factors. Tables S2 and S3 revealed that water depth and temperature were significant in structuring testate amoebae assemblages, respectively. These limnological variables may easily contribute to structuring testate amoebae assemblages either directly or through their association with other biological factors. For example, water temperature may contribute to the distribution of testate amoebae indirectly as it interacts strongly with food availability, phytoplankton abundance, sediment properties, oxygen conditions and optical properties [34, 58, 73]. Furthermore, significant effects of chlorophyll a and TN on *Difflugia* assemblages while temperature for abundance-based communities without *Difflugia* species except geographical factors indicating that rare species may be affected by different environmental variables. Thus, the distribution of testate amoebae was, perhaps unsurprisingly, a complex effect of climatic, geographical and limnological factors along with potential effects from human activities.

### Latitudinal Gradient in Testate Amoebae

Hillebrand and Azovsky concluded that the species richness of large organisms such as vertebrates decreases from the tropics to the poles, whereas the relatively small protozoa showed weak or no correlations between species richness and latitude [31]. For testate amoebae, there has to our knowledge been only one report describing a decrease in species richness towards high southern latitudes from temperate to polar regions [66]. We expected to find either a negative correlation between latitude and testate amoebae diversity in lake ecosystems or no correlation, but our results did not appear to support either of these alternatives. On the contrary, a remarkable reversed trend was found with taxon richness increasing at higher latitudes. Such a pattern would be unusual, but not unique, as various macroscopic taxa—such as penguins, and both soil-living oribatid mites and nematodes showed such a pattern [64].

An obvious confounding factor in our data is the non-random altitudinal distribution along our transect—for example some of the mid-latitude lakes are at over 4,000 m while many of the high-latitude ones are around 130 m in altitude. A simple approach to removing this effect is to analyze a sub-sample of the lakes of similar altitude. Lakes below 150 m (*n* = 25) are found throughout our transect, and for these, there is no significant correlation between latitude and species richness (Fig. S3). Another more complex approach is to reassign all the lakes’ new latitudes based on their altitude values. Again, the significant correlation between species richness and latitude vanished (*n* = 51), neither does abundance-based Shannon-Wiener index nor does biomass-based Shannon-Wiener index (Fig. S4). Thus, we suspect that the interesting ‘reverse’ relationship between species richness and latitude is an artifact of the altitude distribution of our sampling sites.

For testate amoebae, a close correlation between species richness and climate variables has previously been reported [80, 87]. In this study, the water temperature did not manifest a conspicuous relationship with testate amoebae species richness along latitude (*r*_s_ = 0.085, *P* = 0.551, *n* = 51). In fact, the seasonal temperature variation was great especially in higher latitude, and the range of mean temperature between July and January was significantly positively correlated with testate amoebae Shannon-Wiener indices and species richness (*P* < 0.001, *n* = 51). Normally, the mean temperature of the regional atmosphere is highly correlated with the water temperature in the epilimnion layers. Although all samples were collected during the summer, the water temperature still varied from 7.10 to 33.04 °C, which largely contributed to the distribution and diversity of testate amoebae. Broader temperature range may help to select or harbour more species which could live in cold climate conditions according to their unique niche width. For example, Patterson et al. noted that the division between two recognized testate amoebae assemblages appears to be a function of seasonal temperatures (assemblage I occurs where summer temperatures reach values greater than 18 °C while assemblage II occurs below the thermocline where temperatures do not exceed 6 °C) [51]. Further, wider temperature ranges could increase habitat heterogeneity by changing the diversity of aquatic macrophytes or other organisms, and thereby the testate amoebae species richness.

Furthermore, White et al. showed that zooplankton biomass generally increased in the epipelagic zone (0–200 m) towards the equator in the Pacific Ocean roughly along 140° W from 12° N to 12° S [79]. This increasing trend towards lower latitude in zooplankton biomass was consistent with our results. The growth rate of organisms was presumably directly, or indirectly, affected by energy or temperature [34, 58]. In our study, the majority of testate amoebae were planktonic forms [85, 87, 89]. Low temperature in winter in higher latitude could largely limit the growth of aquatic organisms including testate amoebae and their food (e.g. algae) [73], resulting in less biomass compared with those in the lower latitude. Thus, the decreasing biomass of testate amoebae with increasing latitude may be due to the temperature variation—note that our sediment samples represented the last 1–4 years’ lake production [68] and so are an ‘average’ of several years’ production. Since the abundance of testate amoebae was positively related to the biomass in this study, it was not surprising that abundance displayed the similar distribution trend with latitude as biomass did.

Comparisons of abundance, biomass and diversity of testate amoebae in lake sediments along latitudinal gradients would ideally include tropical, temperate and Antarctic/Arctic zones. In fact, studies on bacteria or metazoa along latitudinal gradient that span the three climate zone have been conducted. For instance, Fuhrman et al. found that a significant latitudinal gradient in planktonic marine bacteria richness from tropic to polar in both hemispheres with maximum appeared in lower latitude [24]. However, Procter found that the species richness, densities and biomass of free-living soil nematodes were often higher at high latitudes than at lower latitudes and nematodes were most diverse and abundant in temperate regions [54]. One limitation of our research was that our study area covered mostly temperate and subtropical regions. Overall, our results provided basic ecological data on the relationship between testate amoebae and latitude and indicated that temperature may be an important predictor of differences in community composition of testate amoebae.

### Trophic Gradient in Testate Amoebae

The response of testate amoebae to trophic status has been reported in lakes and rivers from many different regions [6, 10], and the results suggested that testate amoebae could be a new tool for monitoring long and short terms of trophic status changes in aquatic ecosystems. Yang et al. revealed that some *Centropyxis* species were rare in hypereutrophic lakes [86], which was in line with our results which showed that *C. playstoma* only dominate in lakes with comprehensive TSI below 60, namely below middle eutrophic level. Moreover, Qin et al. suggested that *Difflugia biwae*, *Difflugia tuberspinifera* and *Difflugia pristis* were good indicators of oligotrophic conditions while *Difflugia oblonga*, *Difflugia corona*, *Difflugia smilion* and *Difflugia lanceolata* were species more common to mesotrophic and eutrophic systems [55], which was also in accordance with our results that *D. biwae* and *D. tuberspinifera* mainly occurred at light eutrophic and middle eutrophic levels. Additionally, the occurrence of *Arcella hemisphaerica* and *Centropyxis aerophila* indicated oligotrophic conditions, and the appearance of *Difflugia urceolata* indicated significant eutrophication [6]. It appeared that some testate amoebae species may have a preference for certain trophic levels; thus, the community composition can be used as indicators of extreme living environment or rapid changes of lake ecosystems.

The abundance and biomass of testate amoebae in our study did not manifest a unimodal relationship with trophic status as we have hypothesized. Given the discussion of the latitude gradient above, one possible reason is that the testate amoebae abundance and biomass were affected more by temperature variation. However, our results suggest that a unimodal relationship between testate amoebae diversity and trophic status with the highest diversity occurred in lakes of mid-eutrophic status. That is, most of testate amoebae species would prefer moderate trophic status, while only few species may prefer extreme environment, either oligotrophic or hypereutrophic. This relationship is also well known from many terrestrial plant communities [27, 28]. The number of samples involved in our studies was of the same order of magnitude as that of many other studies for which unimodal responses have been reported [19]; thus, the trophic status gradient spanned from oligotrophic to hypereutrophic status, and the range was wide enough to detect the unimodal curve. Many studies of the historical development in diversity of diatoms or zooplankton also revealed a unimodal relationship with increased trophic status [19, 33], whereas others record a monotonical decline [39].

Diversity indices were sensitive to invasion barriers, pH, latitude, lake area, habitat heterogeneity, predation and disturbance apart from ecological stress factors such as eutrophication [33]. Thus, microorganism diversity could exhibit various trends along trophic status gradient considering all the affected variables. One of the possible explanations may be that the middle eutrophic conditions provided more appropriate food (mainly bacteria, fungi or protists) for most of testate amoebae species [44, 59]. Simultaneously, high nutrients usually result in changes in community structure at each trophic level or within different taxonomic groups, such as promoting the propagating of algae [40], which may affect the abundance of testate amoebae since algae was one of the important food sources [73]. However, hypereutrophic conditions may lead to harmful algal blooms [2], causing a variety of water quality problems and decreasing testate amoebae abundance and species richness. Further, variables such as heterogeneity and pH may, in turn, be influenced by eutrophication, reflecting changes in amount of their food or predator in a more indirect way, which may provide an additional explanation for unimodal richness responses along broad trophic status gradient in aquatic ecosystems [19, 33]. As a whole, our results provide further support for the idea that testate amoebae diversity and distribution were sensitive to trophic status and maximum diversity appeared in middle eutrophic status.

## Conclusion

This is the most geographically extensive study focusing on testate amoebae communities in the surface lake sediments of China in relation to latitude and trophic status to date. The diversity of testate amoebae in China is high with 169 taxa belonging to 24 genera identified in this study. The diversity and distribution of testate amoebae varied significantly between lakes and reservoirs. However, *Difflugia*, *Centropyxis* and *Arcella* were the most diverse and dominant genera in these freshwater ecosystems—so illustrating Hutchinson’s point about sets of lakes exhibiting an interesting mix of generality and site-specific details [32]. Our results clearly showed that trophic status is significantly related to testate amoebae diversity and distribution. The unimodal relationships between testate amoebae diversity and trophic status indicated that most testate amoebae species are more likely to live in middle eutrophic status environment. The results also appeared to show a significant relationship between species richness and latitude; however, this is probably an artifact of the non-random distribution of altitudes of our study sites. Our results demonstrate that water temperature variation can significantly influence the diversity of testate amoebae community, thereby indicating that testate amoebae could be used as a bioindicator in assessing the global warming.

## Electronic supplementary material

Below is the link to the electronic supplementary material.

Fig. S1 Frequencies of 169 testate amoeba taxa in 51 lakes and reservoirs in China. (DOC 311 kb)

Fig. S2 Redundancy analysis (RDA) sample-environment biplot for the 51 lakes and reservoirs that yield statistically significant testate amoeba populations. (DOC 1916 kb)

Fig. S3 Variation of testate amoeba community parameters along a latitudinal gradient (25 lakes below 150 m height). (DOC 697 kb)

Fig. S4 Variation of testate amoeba community parameters along the corrected latitudinal gradient. (DOC 602 kb)

Table S1 List of the dominant species and subspecies in 51 study lakes and reservoirs of China. (DOC 81 kb)

Table S2 Marginal effect of 9 environment variables based on testate amoeba abundance and biomass data in pCCA. (DOC 39 kb)

Table S3 Marginal effect of 9 environment variables based on testate amoebae in pRDA. (DOC 53.5 kb)

## References

[1] Alves GM, Velho LF, Simoes NR, Lansac-Toha FA (2010). Biodiversity of testate amoebae (Arcellinida and Euglyphida) in different habitats of a lake in the Upper Parana River floodplain. Eur J Protistol.

[2] Anderson D, Glibert PM, Burkholder JM (2002). Harmful algal blooms and eutrophication: nutrient sources, composition, and consequences. Estuaries.

[3] Arnell NW (1999). Climate change and global water resources. Global Environ Chang.

[4] Beyens L, Chardez D, Baere DD, Verbruggen C (1995). The aquatic testate amoebae fauna of the Strømness Bay area, South Georgia. Antarct Sci.

[5] Beyens L, Chardez D, Landtsheer RD, Baere DD (1986). Testate amoebae communities from aquatic habitats in the Arctic. Polar Biol.

[6] Beyens L, Meisterfeld R (2001) Protozoa: testate amoebae. In: Smol JP, Birks HJB, Last WM (ed) Tracking environmental change using lake sediments. Kluwer Academic Publishers, Dordrecht. 3:121–153

[7] Bobrov A, Mazei Y, Chernyshov V, Gong YC, Feng WS (2012). Testate amoebae communities from some freshwater and soil habitats in China (Hubei and Shandong Provinces). Front Earth Sci China.

[8] Bobrov A, Qin Y, Wilkinson DM (2014) Latitudinal diversity gradients in free-living microorganisms— *Hoogenraadia* a key genus in testate amoebae biogeography. Acta Protozool In press

[9] Brooks SJ, Bennion H, Birks HJB (2001). Tracing lake trophic history with a chironomid–total phosphorus inference model. Freshwat Biol.

[10] Burbidge SM, Schröder-Adams CJ (1998). Thecamoebians in Lake Winnipeg: a tool for Holocene paleolimnology. J Paleolimnol.

[11] Cai QH (1997). On the comprehensive evaluation methods for lake eutrophication. J Lake Sci.

[12] Carlson RE (1977). A trophic state index for lakes. Limnol Oceanogr.

[13] Cash J, Hopkinson J (1905). The British freshwater Rhizopoda and Heliozoa. Vol. I: Rhizopoda, part I.

[14] Cash J, Hopkinson J (1909). The British freshwater Rhizopoda and Heliozoa. Vol. II: Rhizopoda, part II.

[15] Cash J, Wailes GH, Hopkinson J (1915). The British Freshwater Rhizopoda and Heliozoa. Vol. III: Rhizopoda, part III.

[16] Cash J, Wailes GH, Hopkinson J (1919). The British freshwater Rhizopoda and Heliozoa. Vol. IV: Rhizopoda, part IV.

[17] Chapin FS, Zaveleta ES, Eviner VT, Naylor RL, Vitousek PM, Lavorel S, Reynolds HL, Hooper DU, Sala OE, Hobbie SE, Mack MC, Diaz S (2000). Consequences of changing biodiversity. Nature.

[18] Charman DJ (2001). Biostratigraphic and palaeoenvironmental applications of testate amoebae. Quat Sci Rev.

[19] Declerck S, Vandekerkhove J, Johansson L, Muylaert K, Conde-Porcuna JM, Van Der Gucht K, Perez-Martinez C, Lauridsen T, Schwenk K, Zwart G, Rommens W, Lopez-ramos J, Jeppesen E, Vyverman W, Brendonck L, De Meester L (2005). Multi-group biodiversity in shallow lakes along gradients of phosphorus and water plant cove. Ecology.

[20] Dudgeon D (2010). Prospects for sustaining freshwater biodiversity in the 21st century: linking ecosystem structure and function. Curr Opin Environ Sustain.

[21] Ellison RL (1995). Paleolimnological analysis of Ullswater using testate amoebae. J Paleolimnol.

[22] Escobar J, Brenner M, Whitmore TJ, Kenney WF, Curtis JH (2008). Ecology of testate amoebae (thecamoebians) in subtropical Florida lakes. J Paleolimnol.

[23] Figueredo CC, Giani A (2001). Seasonal variation in the diversity and species richness of phytoplankton in a tropical eutrophic reservoir. Hydrobiologia.

[24] Fuhrman JA, Steele JA, Hewson I, Schwalbach MS, Brown MV, Green JL, Brown JH (2008). A latitudinal diversity gradient in planktonic marine bacteria. Proc Natl Acad Sci U S A.

[25] Gaston KJ (2000). Global patterns in biodiversity. Nature.

[26] Greenberg AE, Clesceri LS, Eaton AD (1992). Standard methods for the examination of water and wastewater.

[27] Grime JP (1973). Competitive exclusion in herbaceous vegetation. Nature.

[28] Grime JP (2001). Plant strategies, vegetation processes and ecosystem properties.

[29] Gunten L, Heiri O, Bigler C, Leeuwen J, Casty C, Lotter AF, Sturm M (2008). Seasonal temperatures for the past ~400 years reconstructed from diatom and chironomid assemblages in a high-altitude lake (Lej da la Tscheppa, Switzerland). J Paleolimnol.

[30] Han BP, Wang T, Xu L, Lin QQ, Jin YZ, Dumont HJ (2011). Carnivorous planktonic *Difflugia* (Protista, Amoebina Testacea) and their predators. Eur J Protistol.

[31] Hillebrand H, Azovsky AI (2001). Body size determines the strength of the latitudinal diversity gradient. Ecography.

[32] Hutchinson GE (1964). The lacustrine microcosm reconsidered. Am Sci.

[33] Jeppesen E, Jensen JP, Søndergaard M, Lauridsen T, Landkildehus F (2000). Trophic structure, species richness and biodiversity in Danish lakes: changes along a phosphorus gradient. Freshwat Biol.

[34] Kindlmann P, Schödelbauerová I, Dixon AFG, Storch D, Marquet PA, Brown JH (2007). Inverse latitudinal gradients in species diversity. Scaling biodiversity.

[35] Kumar A, Dalby AP (1998) Identification key for Holocene lacustrine arcellacean (thecamoebian) taxa. Palaeontologia Electronica, http://palaeo-electronica.org/

[36] Kumar A, Patterson RT (2000). Arcellaceans (thecamoebians): new tools for monitoring long- and shprt-term changes in lake bottom acidity. Environ Geol.

[37] Leidy J (1879). Freshwater rhizopods of North America.

[38] Lotter AF, Birks HJB, Hofmann W, Marchetto A (1997). Modern diatom, cladocera, chironomid, and chrysophyte cyst assemblages as quantitative indicators for the reconstruction of past environmental conditions in the Alps. I. Climate. J Paleolimnol.

[39] Marques JC, Pardal MA, Nielsen SN, Jorgensen SE (1997). Analysis of the properties of exergy and biodiversity along anestuarine gradient of eutrophication. Ecol Model.

[40] Mazumder A (1994). Patterns of algal biomass in dominant odd- vs. even-link lake ecosystems. Ecology.

[41] Meisterfeld R, Lee JJ, Leedale GF, Bradbury PC (2002). Order Arcellinida. The illustrated guide to the protozoa.

[42] Meisterfeld R, Lee JJ, Leedale GF, Bradbury PC (2002). Testate amoebae with filopodia. The illustrated guide to the protozoa.

[43] Meriläinen JJ, Hynynen J, Palomäki A, Reinikainen P, Teppo A, Granberg K (2000). Importance of diffuse nutrient loading and lake level changes to the eutrophication of an originally oligotrophic boreal lake: a palaeolimnological diatom and chironomid analysis. J Paleolimnol.

[44] Mitchell EAD, Bragazza L, Gerdol R (2004). Testate amoebae (Protista) communities in *Hylocomium splendens* (Hedw.) B.S.G. (Bryophyta): relationships with altitude, and moss elemental chemistry. Protist.

[45] Mitchell EAD, Charman DJ, Warner BG (2008). Testate amoebae analysis in ecological and paleoecological studies of wetlands: past, present and future. Biodivers Conserv.

[46] Monteith JL, Unsworth MH (2008). Principles of environmental physics.

[47] Moss B (2010). Ecology of freshwaters.

[48] Nagy L, Grabherr G (2009). The biology of alpine habitats.

[49] Ogden CG, Hedley RH (1980). An atlas of freshwater testate amoebae.

[50] Patterson RT, Kumar A (2002). A review of current testate rhizopod (thecamoebian) research in Canada. Palaeogeogr Palaeoclimatol Palaeoecol.

[51] Patterson RT, Mackinnon KD, Scottand DB, Medioli FS (1985). Arcellaceans (‘thecamoebians’) in small lakes of New Brunswick and Nova Scotia: modern distribution and Holocene stratigraphic changes. J Foraminifer Res.

[52] Payne RJ, Mitchell EAD (2009). How many is enough? Determining optimal count totals forecological and palaeoecological studies of testate amoebae. J Paleolimnol.

[53] Penard E (1902). Faune Rhizopodique du Bassin du Léman.

[54] Procter DLC (1984). Towards a biogeography of free-living soil nematodes. I. Changing species richness, diversity and densities with changing latitude. J Biogeogr.

[55] Qin YM, Booth RK, Gu YS, Wang YX, Xie SC (2009). Testate amoebae as indicators of 20th century environmental change in Lake Zhangdu, China. Fundam Appl Limnol.

[56] Rivadeneira MM, Thiel M, González ER, Haye PA (2011). An inverse latitudinal gradient of diversity of peracarid crustaceans along the Pacific Coast of South America: out of the deep south. Glob Ecol Biogeogr.

[57] Roe HM, Patterson RT, Swindles GT (2009). Controls on the contemporary distribution of lake thecamoebians (testate amoebae) within the Greater Toronto Area and their potential as water quality indicators. J Paleolimnol.

[58] Rohde K (1992). Latitudinal gradients in species diversity: the search for the primary cause. Oikos.

[59] Schmidt AR, Girard V, Perrichot V, Schonborn W (2010). Testate amoebae from a Cretaceous forest floor microbiocoenosis of France. J Eukaryot Microbiol.

[60] Schönborn W (1973). Paläolimnologische Studien an Testaceen aus Bohrkernen des Latnjajaure (Abisko-Gebiet; Schwedisch-Lappland). Hydrobiologia.

[61] Schönborn W (1990). Analyse subfossiler Protozoenschalen der Sedimente eines kleinen Waldsees (Kleiner Barsch-See, nördliche DDR). Limnologica.

[62] Schröter D, Wolters V, De Ruiter PC (2003). C and N mineralisation in the decomposer food webs of a European forest transect. Oikos.

[63] Shen YF, Jiang XZ, Shen YF, Gong XJ (1983). Protozoa of the Tibetan Plateau. Aquatic invertebrates of the Tibetan Plateau.

[64] Sherratt TN, Wilkinson DM (2009). Big questions in ecology and evolution.

[65] Smith HG (1982). The terrestrial protozoan fauna of South Georgia. Polar Biol.

[66] Smith HG (1996). Diversity of Antarctic terrestrial protozoa. Biodivers Conserv.

[67] Smith HG, Bobrov A, Lara E (2008). Diversity and biogeography of testate amoebae. Biodivers Conserv.

[68] Smol JP (2008). Pollution of lakes and rivers: a paleoenvironmental perspective.

[69] Spellerberg IF, Fedor PJ (2003). A tribute to Claude Shannon (1916–2001) and a plea for more rigorous use of species richness, species diversity and the ‘Shannon–Wiener’ index. Glob Ecol Biogeogr.

[70] Stomp M, Huisman J, Mittelbach GG, Litchman E, Klausmeier CA (2011). Large-scale biodiversity patterns in freshwater phytoplankton. Ecology.

[71] ter Braak CJF, Šmilauer P (2002). CANOCO reference manual and CanoDraw for Windows user’s guide: software for canonical community ordination (version 4.5).

[72] Tolonen K, Berglund BE (1986). Rhizopod analysis. Handbook of holocene palaeoecology and palaeohydrology.

[73] Torigai K, Schröder-Adams CJ, Burbidge SM (2000). A variable lacustrine environment in Lake Winnipeg, Manitoba: evidence from modern thecamoebian distribution. J Paleolimnol.

[74] Tsyganov AN, Milbau A, Beyens L (2013). Environmental factors influencing soil testate amoebae in herbaceous and shrubby vegetation along an altitudinal gradient in subarctic tundra (Abisko, Sweden). Eur J Protistol.

[75] Velho LFM, Lansac-Tôha FA, Bini LM (2003). Influence of environmental heterogeneity on the structure of testate amoebae (Protozoa, Rhizopoda) assemblages in the plankton of the upper Paraná River floodplain, Brazil. Int Rev Hydrobiol.

[76] Wall AAJ, Magny M, Mitchell EAD, Vannière B, Gilbert D (2010). Response of testate amoebae assemblages to environmental and climatic changes during the Lateglacial-Holocene transition at Lake Lautrey (Jura Mountains, eastern France). J Quat Sci.

[77] Wang SM, Dou HS (1998). China lakes.

[78] Weisse T, Müller H, Pinto-Coelho RM, Schweizer A, Springmann D, Baldringer G (1990). Response of the microbial loop to the phytoplankton spring bloom in a large prealpine lake. Limnol Oceanogr.

[79] White JR, Zhang XS, Welling LA, Roman MR, Dam HG (1995). Latitudinal gradients in zooplankton biomass in the tropical Pacific at 140 ºW during the JGOFS EqPac study: effects of El Niño. Deep-Sea Res II Top Stud Oceanogr.

[80] Wilkinson DM (1994). A review of the biogeography of the protozoan genus *Nebela* in the southern temperate and Antarctic zones. Area.

[81] Willig MR, Kaufmann DM, Stevens RD (2003). Latitudinal gradients of biodiversity: pattern, process, scale and synthesis. Annu Rev Ecol Syst.

[82] Xu ZL, Chen YQ (1989). Aggregated idensity of dominant species of zooplankton in autumn in the East China Sea and Yellow Sea. Chin J Ecol.

[83] Yang J, Smith HG, Sherratt TN, Wilkinson DM (2010). Is there a size limit for cosmopolitan distribution in free-living microorganisms? A biogeographical analysis of testate amoebae from polar areas. Microb Ecol.

[84] Yang J, Yu XQ, Liu LM, Zhang WJ, Guo PY (2012). Algae community and trophic state of subtropical reservoirs in southeast Fujian, China. Environ Sci Pollut Res.

[85] Yang J, Zhang WJ, Feng WS, Shen YF (2005). Testate amoebae (Protozoa: Rhizopoda) from Northwest Yunnan, China. J Freshwat Ecol.

[86] Yang J, Zhang WJ, Feng WS, Shen YF (2005). Freshwater testate amoebae of nine Yunnan Plateau lakes, China. J Freshwat Ecol.

[87] Yang J, Zhang WJ, Feng WS, Shen YF (2006). Geographical distribution of testate amoebae in Tibet and northwestern Yunnan and their relationships with climate. Hydrobiologia.

[88] Yang J, Zhang WJ, Shen YF (2009). Relationships between testate amoebae assemblages (Protozoa) and geographic factors in Yunnan Plateau lakes, China. J Freshwat Ecol.

[89] Yu Z, Zhang WJ, Liu LM, Yang J (2014). Evidence for two different morphotypes of *Difflugia tuberspinifera* from China. Eur J Protistol.

