# Supplementary figures and images for: Diversity and Distribution of Freshwater Testate Amoebae (Protozoa) Along Latitudinal and Trophic Gradients in China
Source: Microb Ecol. 2014 Jun 10;68(4):657–70. doi: 10.1007/s00248-014-0442-1 (PMC4201926; doi:10.1007/s00248-014-0442-1)

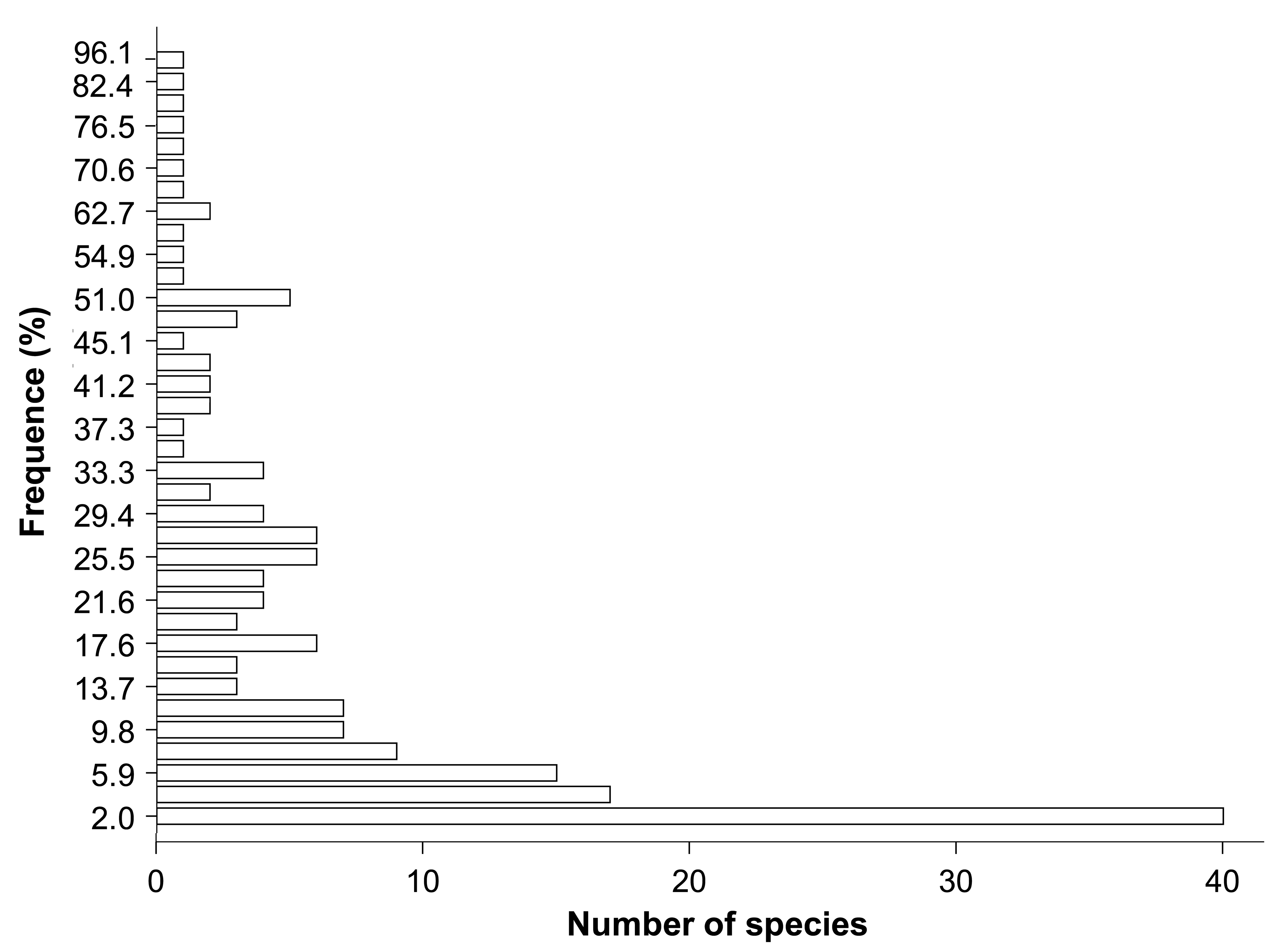


**Fig. S1** Frequencies of 169 testate amoeba taxa in 51 lakes and reservoirs in China

Supplement: Supplementary file 1 — Frequencies of 169 testate amoeba taxa in 51 lakes and reservoirs in China. (DOC 311 kb) [file 248_2014_442_MOESM1_ESM.doc]
