# Supplementary material for: Diversity and Distribution of Freshwater Testate Amoebae (Protozoa) Along Latitudinal and Trophic Gradients in China
Source: Microb Ecol. 2014 Jun 10;68(4):657–70. doi: 10.1007/s00248-014-0442-1 (PMC4201926; doi:10.1007/s00248-014-0442-1)
Supplement: Supplementary file 2 — Redundancy analysis (RDA) sample-environment biplot for the 51 lakes and reservoirs that yield statistically significant testate amoeba populations. (DOC 1916 kb) [file 248_2014_442_MOESM2_ESM.doc]

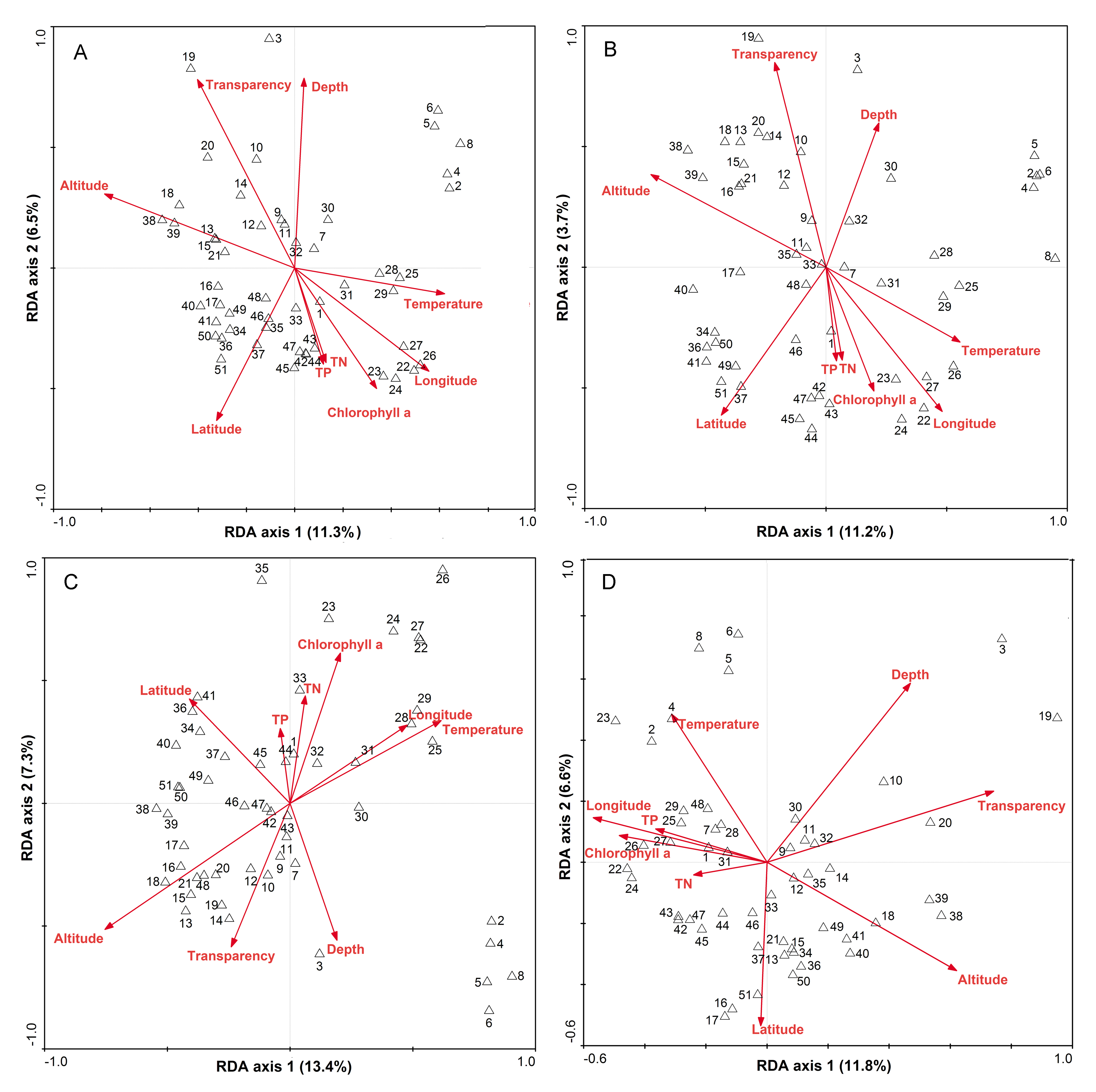


**Fig. S2** Redundancy analysis (RDA) sample-environment biplot for the 51 lakes and reservoirs that yield statistically significant testate amoeba populations. A. all abundance data. B. all biomass data. C. abundance data with only *Difflugia* species. D. abundance data without *Difflugia* species. The 51 sample sites are given in Table 1
