# Supplementary material for: Diversity and Distribution of Freshwater Testate Amoebae (Protozoa) Along Latitudinal and Trophic Gradients in China
Source: Microb Ecol. 2014 Jun 10;68(4):657–70. doi: 10.1007/s00248-014-0442-1 (PMC4201926; doi:10.1007/s00248-014-0442-1)
Supplement: Supplementary file 3 — Variation of testate amoeba community parameters along a latitudinal gradient (25 lakes below 150 m height). (DOC 697 kb) [file 248_2014_442_MOESM3_ESM.doc]

**
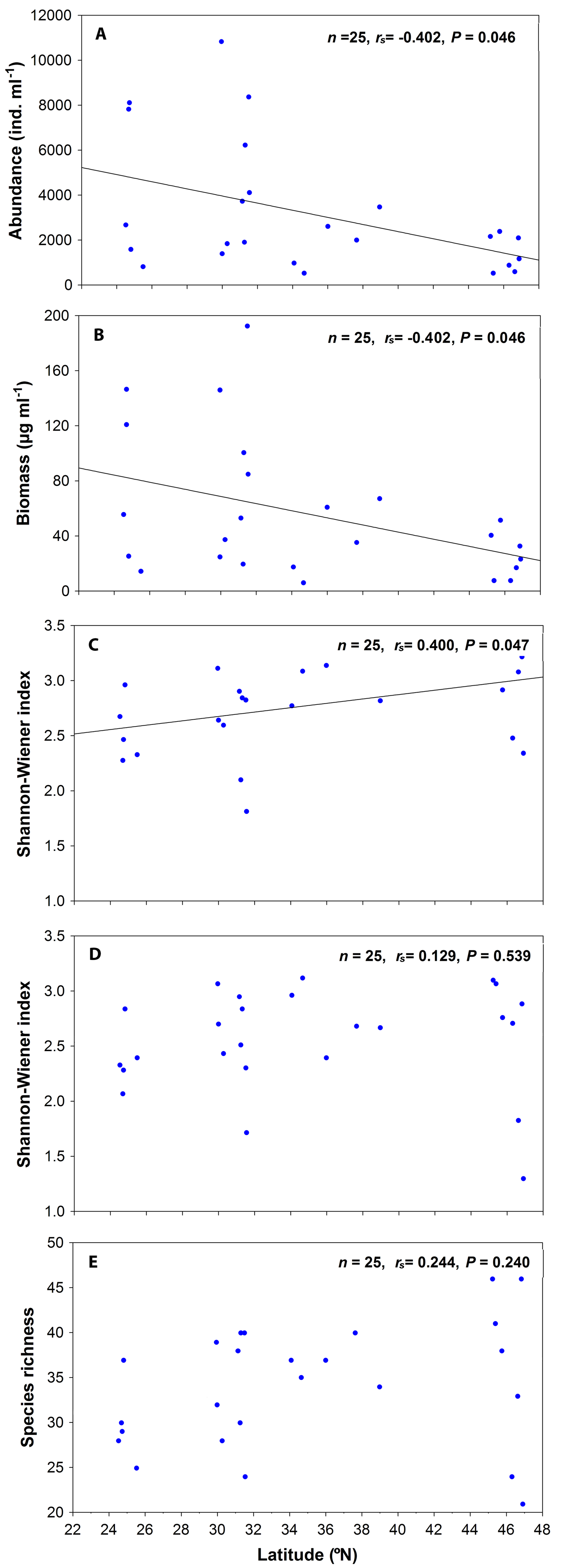
**

**Fig. S3** Variation of testate amoeba community parameters along a latitudinal gradient (25 lakes below 150 m height). **A.** Abundance. **B.** biomass. **C.** Shannon-Wiener index based on abundance data. **D.** Shannon-Wiener index based on biomass data. **E.** species richness
