# Supplementary material for: Diversity and Distribution of Freshwater Testate Amoebae (Protozoa) Along Latitudinal and Trophic Gradients in China
Source: Microb Ecol. 2014 Jun 10;68(4):657–70. doi: 10.1007/s00248-014-0442-1 (PMC4201926; doi:10.1007/s00248-014-0442-1)
Supplement: Supplementary file 4 — Variation of testate amoeba community parameters along the corrected latitudinal gradient. (DOC 602 kb) [file 248_2014_442_MOESM4_ESM.doc]

**
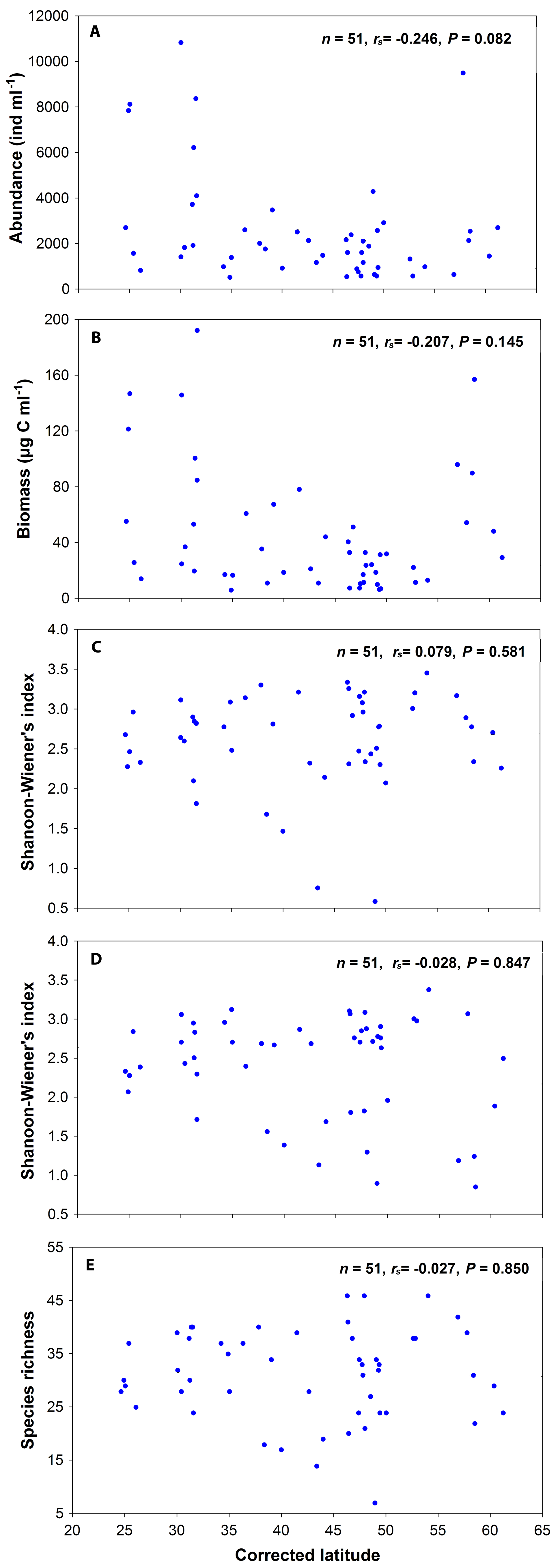
**

**Fig. S4** Variation of testate amoeba community parameters along the corrected latitudinal gradient (i.e. latitude adjusted using standard lapse rates to ‘correct’ for altitude – see text for details and caveats). **A.** abundance. **B.** biomass. **C.** Shannon-Wiener index based on abundance data. **D.** Shannon-Wiener index based on biomass data. **E.** species richness
