# Supplementary material for: Diversity and Distribution of Freshwater Testate Amoebae (Protozoa) Along Latitudinal and Trophic Gradients in China
Source: Microb Ecol. 2014 Jun 10;68(4):657–70. doi: 10.1007/s00248-014-0442-1 (PMC4201926; doi:10.1007/s00248-014-0442-1)
Supplement: Supplementary file 5 — List of the dominant species and subspecies in 51 study lakes and reservoirs of China. (DOC 81 kb) [file 248_2014_442_MOESM5_ESM.doc]

**Table S1 List of the dominant species and subspecies in 51 study lakes and reservoirs of China**

| **Lake code** | **Dominant species** |
| --- | --- |
| YLL | *Centropyxis aculeata, C. aculeata minima, C. aerophila, C. ecornis, Difflugia elegans,*  *D. gramen, D. manicata, D. penardi, D. pristis, D. pulex, Pseudodifflugia gracilis, Zivkovicia compressa* |
| HBR | *C. aculeata minima, D. acuminata, D. elegans, D. gramen, D. gramen globulosa,*  *D. kabylica, D. limnetica, D. mica, D.* sp3*., D. tuberspinifera, Lesquereusia modesta, Pseudodifflugia gracilis* |
| FXL | *Arcella arenaria，A. hemisphaerica, Cyphoderia ampulla, D. penardi, D. pristis* |
| BTR | 1. *hemisphaerica, D. gramen, D. gramen globulosa, D. kabylica, D. limnetica,*   *D. mulanensis, D. tuberspinifera, Pseudodifflugia gracilis* |
| SDR | *D. gramen globulosa, D. kabylica* |
| TXR | *Bullinularia minor, C. aculeata, C. aculeata minima, C. aerophila, C. platystoma,*  *D. gramen globulosa, D. kabylica, D. limnetica, D. mulanensis, D. oblonga oblonga,*  *D. penardi, D. tuberspinifera, Euglypha acanthophora, L. modesta* |
| DCL | *D. limnetica, D. mammillaris, D. penardi, D.* sp5*., D. tuberculata, Z. compressa* |
| DZR | *D. biwae, D. gramen, D. gramen globulosa, D. kabylica, D. limnetica,*  *D. oblonga oblonga, D. tuberspinifera, Pseudodifflugia gracilis* |
| EHL | 1. *discoids, C. aculeata, C. aculeata minima, C. ecornis, C. platystoma armata,*   *C. sylvatica, D. bryophila, D. elegans, D. levanderi, D. oblonga oblonga, D. pristis,*  *D. pulex, Lagenodifflugia bryophila, L. modesta, Pontigulasia incisa, Z.compressa* |
| CBL | 1. *hemisphaerica, D. acuminata, D. elegans, D. gramen, D. gramen globulosa,*   *D. kabylica* |
| HXH | *A. vulgaris, D. gramen* |
| JHL | *C. aculeata, C. aculeata minima, C. aerophila, C. discoides, C. ecornis, C. hirsuta,*  *D. elegans, D. globulosa, D. gramen* |
| SML | *C. aerophila, Cyphoderia ampulla, D. elegans, D. penardi, D. rutunda, D. stoutii,*  *D. viscidula, Euglypha acanthophora, Pseudodifflugia gracilis* |
| ZML | *C. aerophila, C. ecornis, C. platystoma, Cyphoderia ampulla, D. brevicolla, D. difficilis, D. elegans, D. lemani, D. masaruzii, D. minuta, D. oblonga oblonga, D. pristis,*  *D. viscidula, Euglypha acanthophora, Sphenodetia lenta* |
| TCL | 1. *hemisphaerica, Bullinularia minor, C. aerophila, D. angulostoma, D. elegans,*   *D. penardi, D. pristis, D. pulex, Nebela dentistoma, Phryganella acropodia, Pseudodifflugia gracilis, Trinema enchelys* |
| LSH | 1. *hemisphaerica undulata, A. rotundata aplanata, C. aculeata, C. aculeata minima,*   *C. aerophila, C. ecornis, C. hirsuta, C. sylvatica, D. globulosa* |
| HBHU | *B.indica, C. aerophila, C. sphagnicola, C. constricta, C. minuta, C. platystoma,*  *C. sylvatica, D. mica, D. minuta, D. penardi* |
| HBHE | *C. aculeata, C. aerophila, C. platystoma, Cyphoderia ampulla, D. angulostoma, D. mica, D. nodosa, D. oblonga oblonga, D. pristis, D. viscidula* |
| LGUL | 1. *hemisphaerica, A. hemisphaerica undulata, A. rotundata aplanata, Arcella* sp*., Cyphoderia ampulla, D. gramen, D. oblonga oblonga, D. penardi, D. pristis,*   *Z. compresssa* |
| XHZ | *A. arenaria, A. hemisphaerica, A. hemisphaerica undulata, D. limnetica, D. lithophila,*  *D. mammillaris, Hyalosphenia subflava* |
| SDL | *A. vulgaris, C. aculeata minima, C. ecornis, D. acuminata, D. amphoralis, D. elegans,*  *D. gramen, D. lithophila, D. mammillaris, D. oblonga angusticollis, Lagenodifflugia epiouxi, N. dentistoma, Z. compressa* |
| LGAL | *C. aculeata, C. aculeata minima, C. aerophila, D. acuminata, D. difficilis, D. gramen,*  *D. gramen globulosa, D. limnetica, D. manicata, D. penardi, D. pristis, D.* sp3*.* |
| TBL | *D. avellana, D. elegans, D. gramen, D. limnetica, D. manicata, D. paulii, D. penardi,*  *D. pristis, L.modesta，Pseudodifflugia gracilis* |
| LZL | *C. aculeata, C. aculeata minima, D. gassowkii, D. gramen, D. limnetica, D. manicata，*  *D. oblonga oblonga, D.* sp3*., D. tuberspinifera, L. modesta* |
| NYL | *D. acuminata, D. biwae, D. difficilis ecornis, D. elegans, D. gassowkii, D. gramen,*  *D. kabylica, D. lemani, D. limnetica, D. masaruzii, D. penardi, D.* sp3*.,*  *D. tuberspinifera, Pseudodifflugia gracilis* |
| TAL | *D. difficilis ecornis, D. globulosa, D. gramen, D. lemani, D. limnetica, D. pristis, D.* sp3*.* |
| GCL | *C. aculeata, C. aculeata minima, C. aerophila, C. hirsuta, C. minuta, D. elegans,*  *D. gramen, D. kabylica, D. limnetica, D. manicata, D. penardi, Pseudodifflugia gracilis* |
| SJL | *C. aculeata, C. aculeata minima, D. biwae, D. gramen, D. gramen globulosa,*  *D. kabylica, D. limnetica, D. oblonga oblonga, D. penardi, D. pristis, D. tuberspinifera* |
| CHL | *D. difficilis ecornis, D. globulosa, D. limnetica, D. pristis, D.* sp3*., D. viscidula* |
| LML | *C. hirsuta, D. avellana, D. bidens, D. lanceolata, D. lemani, D. minuta,*  *D. oblonga oblonga, D. penardi, D.* sp3*., Lagenodifflugia vas, Phryganella acropodia* |
| WSL | 1. *rotundata aplanata, C. aculeata, C. aculeata minima, C. aerophila, C. cassis,*   *C. hirsuta, Cyclopyxis eurystoma, Cyphoderia ampulla, D. ampullula, D. gramen,*  *D. lemani, D. pristis, D. schuurmani, D.* sp3*., Phryganella acropodia* |
| DPL | *C. aerophila, Cyphoderia ampulla, D. curvicaulis, D. elegans, D. gassowkii, D. gramen, D. manicata, D. oblonga oblonga, D. penardi, D. pristis, D. schuurmani, D.* sp3*., D.* sp4*., D. tuberspinifera, L. modesta, Phryganella acropodia* |
| HSL | *C. aculeata, C. aculeata minima, C. aerophila, C. cassis spinifera, C. ecornis, C. hirsuta, C. orbicularis, D. elegans, D. gassowkii, D. gramen, D. lucida, D. mammillaris,*  *D. minuta, D. penardi, Phryganella acropodia, Phryganella hemisphaerica* |
| YHL | *C. aculeata, C. aerophila, C. cassis, C. ecornis, C. hirsuta, Cyclopyxis eurystoma,*  *D. curvicaulis, D. globulosa, D. gramen, D. limnetica, D. manicata，D. minuta,*  *D. penardi, D. pristis, Phryganella acropodia, Pseudodifflugia gracilis* |
| BYD | *C. aculeata minima, C. aerophila, C. ecornis, C. hirsuta, Cyclopyxis eurystoma,*  *D. bacillariarum, D. lanceolata, D. linearis, D. oblonga oblonga, D. paulii, D.* sp3*., Phryganella acropodia* |
| XHL | *C. aerophila, Cyclopyxis eurystoma, Cyphoderia ampulla, D. ampullula, D. globulosa, D. lemani, D. manicata, D. masaruzii, D. mica, D. minuta, D. penardi, D. pristis,*  *D. pulex, Phryganella acropodia, Pseudodifflugia gracilis* |
| HSH | *C. aculeata, C. aculeata minima, C. aerophila, C. hirsuta, C. orbicularis, D. acuminata, D. elegans, D. gramen, D. penardi, D. schuurmani, Phryganella acropodia* |
| DHZ | *A. bathystoma, A. hemisphaerica, Phryganella acropodia* |
| WLSH | *A. hemisphaerica, C. aculeata, C. aculeata minima, C. aerophila, C. ecornis,*  *D. oblonga oblonga* |
| QSHZ | *A. hemisphaerica, C. aculeata minima, C. aerophila, C. platystoma,*  *Cyclopyxis eurystoma, D. elegans, D. globulosa, D. lanceolata, D. manicata,*  *D. minuta, Phryganella acropodia* |
| SLHZ | *A. hemisphaerica, C. aerophila, D. ampullula, D. globulosa, D. penardi, D. pristis, Phryganella acropodia* |
| XMP | *D. acuminata, D. difficilis, D. gassowkii, D. gramen, D. limnetica, D. lucida,*  *D. mammillaris, D. manicata, D. mica, D. molesta, D. paulii, D. penardi* |
| KLP | *Bullinularia minor, Cyphoderia ampulla, D. acuminata, D. difficilis, D. elegans,*  *D. gramen, D. lemani, D. mammillaris, D. manicata, D. paulii, D. penardi, D. pristis, Phryganella acropodia* |
| YLP | *C. aculeata minima, C. aerophila, D. acuminata, D. difficilis, D. gramen, D. limnetica, D. manicata, D. paulii, D. penardi, D. pristis, Nebela* sp*., Phryganella hemisphaerica* |
| LMSP | *Bullinularia minor, C. aculeata minima,C. aerophila, C. sphagnicola, D. gramen,*  *D. gramen globulosa, D. limnetica, D. minuta, D. penardi, Phryganella acropodia* |
| AMTP | *C. aerophila, Cyclopyxis eurystoma, D. acuminata, D. ampullula, D. gramen,*  *D. hiraethogii, D. levanderi, D. mica, D. minuta, D. penardi, D. pristis, D. tenuis, Phryganella acropodia, Phryganella hemisphaerica, Z.compressa* |
| QJP | *D. acuminata, D. avellana, D. gramen, D. limnetica, D. mammillaris, D. masaruzii,*  *D. mica, D. minuta, D. oblonga oblonga, D. penardi, D.* sp1*., D. tenuis,*  *Pontigulasia incisa, Pseudodifflugia gracilis* |
| TIL | *Bullinularia minor, C. aerophila, C. sphagnicola, C. cassis, C. minuta,*  *Cyclopyxis eurystoma, D. lemani, D. mica, D. minuta, D. penardi, D.* sp2*., Phryganella hemisphaerica* |
| BEL | *A. rotundata, C. aculeata, C. aculeata grandis, C. aculeata minima, C. aerophila,*  *C. ecornis, C. hirsuta, C. minuta, C. percolabiensis inermis, C. platystoma, D. elegans, D. mica* |
| WLP | *C. aculeata, C. aculeata minima, C. aerophila, C. ecornis, C. sylvatica, D. avellana,*  *D. gramen, D. hiraethogii, D. mica, D. penardi, D. pristis, D. pulex* |
| HHNE | *C. aerophila, D. acuminata, D. ampullula, D. difficilis, D. elegans, D. gramen,*  *D. gramen globulosa, D. lacustris, D. limnetica, D. manicata, D. penardi, D. pristis,*  *D. pulex, D. stoutii* |
