# Supplementary material for: Diversity and Distribution of Freshwater Testate Amoebae (Protozoa) Along Latitudinal and Trophic Gradients in China
Source: Microb Ecol. 2014 Jun 10;68(4):657–70. doi: 10.1007/s00248-014-0442-1 (PMC4201926; doi:10.1007/s00248-014-0442-1)
Supplement: Supplementary file 6 — Marginal effect of 9 environment variables based on testate amoeba abundance and biomass data in pCCA. (DOC 39 kb) [file 248_2014_442_MOESM6_ESM.doc]

**Table S2** Marginal effect of 9 environment variables based on testate amoeba abundance and biomass data in pCCA

| **Variable**  **（abundance）** | **Percentage variance (%)** | **Significance**  **level** | **Variable**  **(biomass)** | **Percentage variance (%)** | **Significance**  **level** |
| --- | --- | --- | --- | --- | --- |
| Latitude | 4.2 | 0.006 | Latitude | 3.4 | 0.045 |
| Longitude | 3.5 | 0.023 | Longitude | 2.5 | 0.381 |
| Altitude | 3.8 | 0.017 | Altitude | 2.2 | 0.582 |
| Depth | 3.5 | 0.031 | Depth | 3.7 | 0.029 |
| Temperature | 3.3 | 0.086 | Temperature | 3.5 | 0.071 |
| Transparency | 3.2 | 0.065 | Transparency | 2.9 | 0.198 |
| Chlorophyll-a | 2.4 | 0.410 | Chlorophyll-a | 1.5 | 0.926 |
| TN | 2.9 | 0.148 | TN | 2.3 | 0.513 |
| TP | 3.0 | 0.216 | TP | 2.2 | 0.473 |
