# Supplementary material for: Diversity and Distribution of Freshwater Testate Amoebae (Protozoa) Along Latitudinal and Trophic Gradients in China
Source: Microb Ecol. 2014 Jun 10;68(4):657–70. doi: 10.1007/s00248-014-0442-1 (PMC4201926; doi:10.1007/s00248-014-0442-1)
Supplement: Supplementary file 7 — Marginal effect of 9 environment variables based on testate amoebae in pRDA. (DOC 53.5 kb) [file 248_2014_442_MOESM7_ESM.doc]

**Table S3** Marginal effect of 9 environment variables based on testate amoebae in pRDA

| **Variable** | **Percentage**  **variance (%)** | **Significance level** | **Variable** | **Percentage**  **variance (%)** | **Significance**  **level** |
| --- | --- | --- | --- | --- | --- |
| **all data abundance** | | | **all data biomass** | | |
| Latitude | 6.1 | 0.001 | Latitude | 5.7 | 0.001 |
| Longitude | 5.0 | 0.003 | Longitude | 3.6 | 0.049 |
| Altitude | 5.2 | 0.001 | Altitude | 2.9 | 0.16 |
| Depth | 2.1 | 0.591 | Depth | 3.1 | 0.121 |
| Temperature | 3.6 | 0.049 | Temperature | 3.1 | 0.131 |
| Transparency | 3.1 | 0.105 | Transparency | 3.3 | 0.074 |
| Chl-a | 3.9 | 0.029 | Chl-a | 3.0 | 0.184 |
| TN | 3.7 | 0.033 | TN | 3.3 | 0.065 |
| TP | 2.7 | 0.261 | TP | 2.7 | 0.280 |
| **abundance with only *Difflugia* species** | | | **abundance without *Difflugia* species** | | |
| Latitude | 7.1 | 0.001 | Latitude | 4.8 | 0.020 |
| Longitude | 6.4 | 0.001 | Longitude | 3.1 | 0.193 |
| Altitude | 7.7 | 0.001 | Altitude | 1.7 | 0.793 |
| Depth | 2.1 | 0.591 | Depth | 3.4 | 0.118 |
| Temperature | 2.9 | 0.222 | Temperature | 4.5 | 0.040 |
| Transparency | 2.7 | 0.282 | Transparency | 3.7 | 0.070 |
| Chl-a | 5.1 | 0.004 | Chl-a | 2.3 | 0.497 |
| TN | 4.4 | 0.017 | TN | 2.8 | 0.272 |
| TP | 2.1 | 0.575 | TP | 3.2 | 0.185 |
